# Identification of miRNAs and their target genes in genic male sterility lines in *Brassica napus* by small RNA sequencing

**DOI:** 10.1186/s12870-021-03306-w

**Published:** 2021-11-09

**Authors:** Jianxia Jiang, Pengfei Xu, Yajie Li, Yanli Li, Xirong Zhou, Meiyan Jiang, Junying Zhang, Jifeng Zhu, Weirong Wang, Liyong Yang

**Affiliations:** 1 Crop Breeding and Cultivation Research Institute, Shanghai Academy of Agricultural Sciences, Shanghai, 201403 China; 2 National Key Laboratory of Plant Molecular Genetics, CAS Center for Excellence in Molecular Plant Sciences, Shanghai Institute of Plant Physiology and Ecology, Chinese Academy of Sciences, Shanghai, 200032 China; 3 School of Agriculture and Biology, Shanghai Jiao Tong University, Shanghai, 200240 China

**Keywords:** *Brassica napus*, miRNAs, Genic male sterility, Pollen development, miR159, Silique development

## Abstract

**Background:**

*Brassica napus* is the third leading source of edible oil in the world. Genic male sterility (GMS) lines provide crucial material for harnessing heterosis for rapeseed. GMS lines have been used successfully for rapeseed hybrid production in China. MicroRNAs (miRNAs) play crucial regulatory roles in various plant growth, development, and stress response processes. However, reports on miRNAs that regulate the pollen development of GMS lines in *B. napus* are few.

**Results:**

In this study, 12 small RNA and transcriptome libraries were constructed and sequenced for the flower buds from the fertile and sterile lines of two recessive GMS (RGMS) lines, namely, “6251AB” and “6284AB”. At the same time, 12 small RNA and transcriptome libraries were also constructed and sequenced for the flower buds from the fertile and sterile lines of two dominant GMS (DGMS) lines, namely, “4001AB” and “4006AB”. Based on the results, 46 known miRNAs, 27 novel miRNAs on the other arm of known pre-miRNAs, and 44 new conserved miRNAs were identified. Thirty-five pairs of novel miRNA-3p/miRNA-5p were found. Among all the identified miRNAs, fifteen differentially expressed miRNAs with over 1.5-fold change between flower buds of sterile and fertile lines were identified, including six differentially expressed miRNAs between “4001A” and “4001B”, two differentially expressed miRNAs between “4006A” and “4006B”, four differentially expressed miRNAs between “6251A” and “6251B”, and ten differentially expressed miRNAs between “6284A” and “6284B”. The correlation analysis of small RNA and transcriptome sequencing was conducted. And 257 candidate target genes were predicted for the 15 differentially expressed miRNAs. The results of 5′ modified RACE indicated that *BnaA09g48720D*, *BnaA09g11120D*, and *BnaCnng51960D* were cleaved by bna-miR398a-3p, bna-miR158-3p and bna-miR159a, respectively. Among the differentially expressed miRNAs, miR159 was chosen to analyze its function. Overexpression of bna-miR159 in *Arabidopsis* resulted in decreased seed setting rate, and shortened siliques, illustrating that miR159 may regulate the fertility and silique development in rapeseed.

**Conclusions:**

Our findings provide an overview of miRNAs that are potentially involved in GMS and pollen development. New information on miRNAs and their related target genes are provided to exploit the GMS mechanism and reveal the miRNA networks in *B. napus.*

**Supplementary Information:**

The online version contains supplementary material available at 10.1186/s12870-021-03306-w.

## Background

Male sterility is largely due to the abnormal development of either the sporophytic or gametophytic anther tissues. According to its origin or genetic analysis, male sterility can be divided into cytoplasmic male sterility (CMS) and genic male sterility (GMS) [1]. CMS is caused by the interaction between the mitochondrial genome and nuclear genes [2, 3]. GMS is derived from natural mutations in nuclear genes that control stamen development. GMS mutants always show advantages, such as complete and stable male sterility and no potential negative cytoplasmic effect, compared with most CMS mutants. GMS can be further divided into dominant GMS (DGMS) and recessive GMS (RGMS). In rapeseed, DGMS and RGMS are widely used for hybrid rapeseed production [4]. Multiple-allele DGMS was generally accepted and was proposed by Song et al. in 2005. This model presented multiple alleles in one locus inheritance to explain the fertility heredity of a newly reported DGMS line 609AB. In this model, *Mf*, *Ms*, and *ms* are three alleles at the same locus, with a relationship of *Mf* dominant over *Ms* and *Ms* over *ms*. The recessive allele is for normal fertility. The maintainers and restorers are easily screened. Thus, multiple-allele DGMS are widely used for hybrid rapeseed seed production through the construction of a three-line hybrid system [5, 6].

Additionally, the recessive GMS (RGMS) systems have another distinct advantage. In RGMS systems, most inbred lines can restore their fertility, so hybrids with strong heterosis are easily bred. Several RGMS lines have been successfully commercialized in China, including S45AB [7], 117AB [8], and 9012AB [9]. For S45AB and 117AB, approximately 50% of the fertile plants are required to be artificially removed before hybridizing the rest (50% sterile ones) with restorer lines in hybrid production, because no complete maintainer line is available [10, 11]. However, a three-line hybrid production system was developed for 9012AB [12], and this system has been well documented [10, 13]. The RGMS line 9012AB has been used successfully for rapeseed hybrid production in China. This male sterility was previously thought to be controlled by three independent genes (*BnMs3*, *BnMs4*, and *BnRf*). In 2012, Dong et al. demonstrated a major modification of the sterility inheritance model in 9012A. The modified inheritance model indicated that the male sterility was essentially controlled by two loci (*BnMs3* and *BnRf*). The previously designated *BnMs4* locus was just one allele of *BnRf*; it was then designated as *BnRf*^*a*^, which was designated in addition to *BnRf*^*b*^ (the allele from 9012A) and *BnRf*^*c*^ (the allele from temporary maintainer). The dominance relationship of the three alleles is in the following order: *BnRf*^*a*^ > *BnRf*^*b*^ > *BnRf*^*c*^. The *BnRf* allele-specific molecular markers were identified; these markers would simplify the breeding process involving this RGMS line [14].

Oilseed rape (*Brassica napus*, 2n = 38, AACC), which has low erucic acid and glucosinolate contents, is the third leading source of edible oil worldwide. In recent years, some conserved and novel miRNAs associated with silique length [15], thickness of pod canopy [16], cadmium stress [17–19], flower organ development [20], seed maturation [21, 22], cold stress [23], seed development, and oil synthesis [24] have been widely identified in rapeseed. However, little information is available about the DGMS and RGMS occurrence at the post-transcriptional level in rapeseed.

In this study, to systematically explore the roles of miRNAs and their targets involved in GMS occurrence during pollen development in rapeseed, 12 small RNA and transcriptome libraries were constructed and sequenced for the flower buds from the fertile and sterile lines of two RGMS lines (“6251AB” and “6284AB”). Meanwhile, 12 small RNA and transcriptome libraries were also constructed and sequenced for the flower buds from the fertile and sterile lines of two DGMS lines (“4001AB” and “4006AB”). The aims of this study were to identify known and potential novel miRNAs from the 24 libraries and to analyze the expression profiles of the miRNAs and their targets in relation to DGMS and RGMS during rapeseed microspore development. The results would provide a foundation for evaluating the important regulatory roles of miRNAs in pollen formation and GMS occurrence in rapeseed and other crops.

## Results

### Analysis of small RNA library data sets and the small RNA profile

**Fig. 1:**
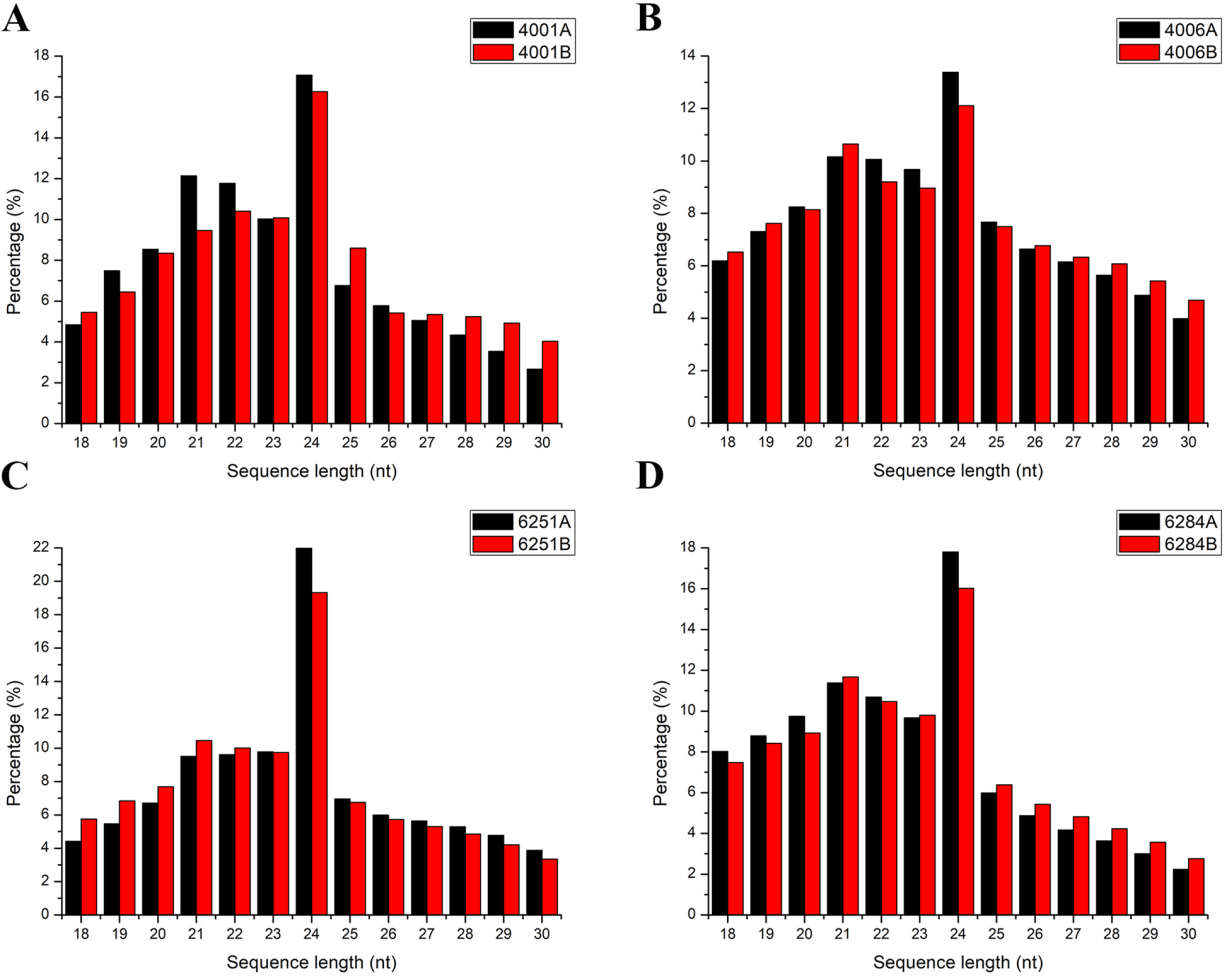
Length distribution of sRNAs in flower buds of A line and B line libraries of DGMS and RGMS in *Brassica napus*. **A** Length distribution of sRNAs in 4001A and 4001B. **B** Length distribution of sRNAs in 4006A and 4006B. **C** Length distribution of sRNAs in 6251A and 6251B. **D** Length distribution of sRNAs in 6284A and 6284B

To identify miRNAs related to DGMS and RGMS during pollen development, the flower buds were collected from the sterile (6251A, 6284A) and fertile (6251B and 6284B) lines of the RGMS lines. Meanwhile, the flower buds were also respectively collected from the sterile (4001A, 4006A) and fertile (4001B and 4006B) lines of the DGMS lines. Three biological replicates were conducted for each of the eight kinds of samples. Thus, total of 24 sRNA libraries were constructed and deep-sequenced. The raw reads of the 24 sRNA libraries ranged from 20.58 to 42.68 million (Table 1). The raw reads of the 24 sRNA libraries were uploaded to SRA database of NCBI and 24 accession numbers were obtained, including SRX11350295, SRX11350296, SRX11350307, SRX11350312, SRX11350313, SRX11350315, and SRX11350316 (https://dataview.ncbi.nlm.nih.gov/object/PRJNA743414?reviewer=t674c02cj415380e8oldre4s5a). After removing the low-quality reads and contaminated adapter sequences, the clean reads of the 24 sRNA libraries ranged from 19.77 to 41.61 million. The mapped reads were further annotated against the Pfam database and subsequently divided into rRNAs, tRNAs, snRNAs, snoRNAs, ta-siRNA, and others. The endogenous sRNAs were identified as known and novel miRNAs. The average sRNA lengths of the three biological replicates for each sample were calculated, which showed the length distribution patterns of the sRNAs being similar to one another. In general, the majority of the small RNAs ranged from 21 nt to 24 nt in size. The 24 nt small RNAs were the most dominant, followed by 21 nt small RNAs (Fig. 1).

**Table 1:** Overview of sRNA sequencing reads in *Brassica napus*

Sample	Raw reads	N% > 10%	Low quality	5^,^ adapter contamine	3^,^ adapter null or insert null	With ployA/T/G/C	Clean reads
4001B-1	24,481,718	41	16,955	8590	761,698	14,436	23,679,998
4001B-2	27,695,084	70	36,718	12,896	698,693	16,785	26,929,922
4001B-3	20,576,690	38	16,651	10,078	768,766	9577	19,771,580
4006B-1	28,457,208	131	44,382	14,481	721,967	14,232	27,662,015
4006B-2	27,355,185	45	7473	9466	895,573	11,463	26,431,165
4006B-3	35,841,966	57	25,238	16,152	1,597,289	18,301	34,184,929
6251B-1	22,307,540	36	18,341	18,038	533,864	14,517	21,722,744
6251B-2	24,409,491	46	15,603	27,336	689,062	7177	23,670,267
6251B-3	24,485,022	34	10,518	24,084	477,366	16,924	23,956,096
6284B-1	25,552,984	64	12,764	21,674	567,970	12,962	24,937,550
6284B-2	24,763,779	34	16,161	23,880	431,298	15,913	24,276,493
6284B-3	25,378,477	40	16,388	25,065	1,546,184	6044	23,784,756
4001A-1	21,968,633	56	16,757	10,355	723,740	8680	21,209,045
4001A-2	26,445,702	23	5505	11,592	1,192,819	7631	25,228,132
4001A-3	27,397,803	96	35,289	12,605	1,483,390	12,457	25,853,966
4006A-1	23,437,578	357	10,961	9403	612,982	14,712	22,789,163
4006A-2	42,675,389	50	10,345	14,879	1,018,244	21,106	41,610,765
4006A-3	27,452,096	93	40,026	12,496	749,916	14,657	26,634,908
6251A-1	21,484,268	19	17,038	20,165	462,483	17,972	20,966,591
6251A-2	24,127,081	56	12,533	20,730	464,608	24,999	23,604,155
6251A-3	21,608,183	357	7763	13,126	393,936	10,376	21,182,625
6284A-2	23,525,443	27	11,236	20,073	746,946	12,136	22,735,025
6284A-2	27,605,112	37	14,315	23,792	1,211,985	12,779	26,342,204
6284A-3	29,689,151	56	30,879	21,998	753,037	16,533	28,866,648

### Identification of known and novel miRNAs in *B. napus*

To identify known miRNAs in *B. napus*, all mapped small RNA sequences were compared with the known mature bna-miRNA sequences deposited in the miRBase database 22.1. Forty-six small RNAs that have the same sequences with the known bna-miRNAs in miRBase were identified. The numbers of reads of the 46 known miRNAs in 24 libraries were listed in Additional Table S1. Among the 46 known miRNAs, bna-miR159a, bna-miR166a, and bna-miR167c showed very high expression levels.

To predict novel miRNAs in *B. napus*, BLAST analysis was conducted for all the mapped small RNAs to the *B. napus* genome sequence in *Brassica* database and known plant miRNAs in miRBase. The small RNAs that exactly map to the genome sequence but not the known plant miRNAs were classified as candidate novel miRNAs. Five criteria described in the Materials and Methods were used to search for novel miRNAs. As a result, 35 pairs of novel miRNA-3p/miRNA-5p were identified. The mature sequences, reads numbers, positions in chromosomes, precursor sequences and minimum free energy were listed in Table 2. The length distribution of the novel miRNAs was between 18 nt to 26 nt. The length of the novel miRNA precursors ranged from 51 nt to 300 nt with an average length of 154 nt. The minimum free energy ranged from − 240.26 to − 9.9 kcal mol^− 1^ with an average of − 70.14 kcal mol^− 1^. The precursor sequences and secondary structures of the novel miRNA were shown in Additional file 1 (Table S2) and Additional file 2 (Fig. S1).

**Table 2:** Novel miRNAs identified in the fertile and sterile lines of *Brassica napus* by high-throughput sequencing

miR_ name	Sequence	Len	Read	pre-position	pre-len	MFE
bna-novel_1-3p	CUUCCUCCUAACACCAAUUGAUU	23	67	chrA09:27889716..27889818	102	−34.6
bna-novel_1-5p	AUCAAUUGGUUUUAGGUUAAGAAGCC	26	124			
bna-novel_2-3p	UGGCAUUGGUAGUAAUGAGUGU	22	190	chrC04:42905032..42905108	76	−26.9
bna-novel_2-5p	ACUCAUUACCAUCAGAGCCAC	21	7			
bna-novel_3-3p	UCAAUGUUGGCUCAAUUAUGU	21	120	chrC02:22515980..22516065	85	−29.5
bna-novel_3-5p	UCAUUGAGUGCAGCGUUGAUGU	22	12			
bna-novel_4-3p	AUUAUCGACACUGAUCUCAUC	21	106	chrC08:2975781..2975915	134	−80.8
bna-novel_4-5p	UAAGGUCACUGUGGUAAUCC	20	52			
bna-novel_5-3p	UCAAUGUUGGCUCAAUUAUG	20	49	chrC09:40959623..40959707	84	−31.6
bna-novel_5-5p	UCAUUGAGUGCAGCGUUGAUGU	22	12			
bna-novel_6-5p	AAGGACUCUAAUCAGAAAUAUUGG	24	143	chrC06:35249139..35249190	51	−9.9
bna-novel_6-3p	AAUGGUCUUAUCUGGAAUCCUUAA	24	11			
bna-novel_7-5p	UGCCUGGCUCCCUGUAUACCA	21	83	chrA08:8293061..8293144	83	−32.4
bna-novel_7-3p	GUGUAUAGAGUAGUCAAGCAUG	22	2			
bna-novel_8-5p	AUCUCUAAUGUAUAACUCCAUUUU	24	24	chrA03:20065617..20065865	248	−95.7
bna-novel_8-3p	AAUGGAGUAGAUAUGGAGAUGCCC	24	1			
bna-novel_9-3p	UUGGACUGAAGGGAACUCCCU	21	1527	chrA09:14645700..14645869	169	−64.4
bna-novel_9-5p	AGAGUUUCCUUAAGUCCAUUC	21	17			
bna-novel_10-5p	UAAGAUCUUUGUACUUUCGGG	21	67	chrA10:15442817..15442916	99	−39.8
bna-novel_10-3p	CGAAAGUACAAAGAUCUGAAA	21	3			
bna-novel_11-5p	AACAGUUGGAUUGGCUCUACGUGG	24	27	chrA09_random:3088720..3089020	300	−65.5
bna-novel_11-3p	ACGAUGGAGGACAAAACUGAUGCA	24	2			
bna-novel_12-5p	UUUUCAGCAAUCUCUUUUCCAUU	23	44	chrA05_random:416209..416319	110	−29.5
bna-novel_12-3p	AUGGGAAAGAUUGUUGAUCAGA	22	6			
bna-novel_13-5p	UAAAGUAGAGCUCGGUGACGG	21	1163	chrC03:20992442..20992727	285	−240.3
bna-novel_13-3p	GUCACCGAGCUCUACUUUAUA	21	1058			
bna-novel_14-5p	UCGCUUCUGUUGAAUAAUUUUGAC	24	22	chrC04:45707769..45708016	247	− 148
bna-novel_14-3p	CAAAAUUAUUCAACAGAAGCGAAU	24	23			
bna-novel_15-5p	AUAUGAGGGUACAAUAGGAAG	21	137	chrAnn_random:33598332..33598540	208	−139.1
bna-novel_15-3p	UAUUGUACCCUCAUAUAUAGC	21	89			
bna-novel_16-3p	CUAAGAGAUCUGUAAUAAACAUGC	24	30	chrC04:7124159..7124378	219	−117.5
bna-novel_16-5p	AUGUUUAUUGUAGGUCUUUUAGGUU	25	7			
bna-novel_17-5p	ACGAACACUGAGUAAUAUCUG	21	15	chrC01_random:3928762..3929012	250	−164.8
bna-novel_17-3p	GAUAUUACUCAGUGUUCGUUG	21	13			
bna-novel_18-3p	ACACUGCAGUGCACUGUACAUUGC	24	17	chrCnn_random:79954335..79954585	250	−113.8
bna-novel_18-5p	GUUGUACAUUGUACACAGCGGUGUAC	26	3			
bna-novel_19-5p	UUGCAAACUGAAUUAUGAGUC	21	20	chrA09:30966144..30966235	91	−45.4
bna-novel_19-3p	CUCAUAAUUCAGUUUGCAAUC	21	20			
bna-novel_20-5p	AAGAUACGGUCUCUUAACUUUUAG	24	259	chrC04_random:3818028..3818149	121	−67.8
bna-novel_20-3p	GUUAAUAGACCGUAUCUUAUA	21	13			
bna-novel_21-5p	AACGAUCUUGUUUGGUUUUGAAGA	24	18	chrA05:21620032..21620188	156	−82.1
bna-novel_21-3p	UUCAAAACCAUACAAGAUCGUUUU	24	24			
bna-novel_22-3p	GAUCAUGUUCGUAGUUUCACC	21	445	chrCnn_random:35712007..35712108	101	−47.3
bna-novel_22-5p	UGAAGCUGCCAGCAUGAUCU	20	3			
bna-novel_23-3p	UUCUUGUGCGUUUAUAGGUAG	21	55	chrA06:23040126..23040236	110	−52.8
bna-novel_23-5p	ACCUCUAAAACACACAAGAAGA	22	3			
bna-novel_24-5p	UGUUUCGCUGUUACUCAUGC	20	40	chrC02:8973302..8973545	243	−93.1
bna-novel_24-3p	AUGAGUAACAGCGAAACAAA	20	26			
bna-novel_25-3p	AAACUGUGUGAACUCUCCAUGGAG	24	389	chrC02_random:2214651..2214881	230	−73.8
bna-novel_25-5p	CCAUAAAAAGGGUUCACAAAGUAUUU	26	1			
bna-novel_26-3p	UUGAUACAUGUAGCUCUUUG	20	2089	chrA03:669098..669269	171	−83.8
bna-novel_26-5p	AAGUGCUACCGGUAUCCACGUG	22	840			
bna-novel_27-5p	UUAAUCGUUUUGUGACUCUU	20	244	chrA07:19146929..19147018	89	−34.7
bna-novel_27-3p	UAGUUACAAAACGAUUAGUGC	21	24			
bna-novel_28-3p	AUCAACGUUGGCUCAAUUAUG	21	453	chrA10_random:1861693..1861781	88	−33.2
bna-novel_28-5p	UCAUUGAGUGCAGCGUUGAUGU	22	12			
bna-novel_29-3p	UCUUGUUACUGAGCUCGACG	20	308	chrA02:7209993..7210285	292	−99.9
bna-novel_29-5p	UUCAGCUGGGUACGAGCCACC	21	710			
bna-novel_30-5p	AUCUGCAUCGAGUGAACUCUAUGG	24	426	chrCnn_random:65177977..65178226	249	−72.9
bna-novel_30-3p	AUGGAAUUCACUGAUGCAGAUGCU	24	7			
bna-novel_31-5p	UUCUUGUGGUUGUAGAGUCUUG	22	367	chrA06:4069612..4069740	128	−56.1
bna-novel_31-3p	AGACUCUACAACAUCAGAAAC	21	47			
bna-novel_32-5p	CGGAUUUUAGCUGCGUAGCUA	21	322	chrAnn_random:44030328..44030409	81	−42.5
bna-novel_32-3p	GGCUACGCUGCUGAAUCCGC	20	2			
bna-novel_33-3p	UUGUAGAAUUUUGGGAAGGGC	21	289	chrC05_random:138762..138826	64	−32.6
bna-novel_33-5p	CCUUCCCAAAAUUCUACAAUU	21	39			
bna-novel_34-5p	ACUUUGAAACUUUGAUCUAGA	21	5292	chrC06:5179422..5179524	102	−42.4
bna-novel_34-3p	UAGAUCAAAGCUUUAAUGU	19	20			
bna-novel_35-3p	UUUUCGAUCUGUAAAUUU	18	4	chrA03:11978303..11978381	78	−30.5
bna-novel_35-5p	CAUUUACAGAUCGAAGACAUU	21	3			

*miR_name* miRNA name, *Len* length of mature miRNA, *pre-position* the position of miRNA precursor sequences in chromosomes of *Brassica napus*, *pre-len* length of miRNA precursor sequences, *MFE* minimum folding free energy, *Read* the total read count of all the small RNA libraries

### Novel miRNA on the other arm of known pre-miRNA

Through sRNA high-throughput sequencing, miRNA-3p and miRNA-5p were found to always be simultaneously present on the 5′ arm and 3′ arm of pre-miRNA secondary structures. To identify novel miRNAs on the other arm of known pre-miRNAs, all mapped small RNA sequences were compared to the known precursor sequences of bna-miRNAs in the miRBase database 22.1. Finally, 27 novel miRNAs on the other arm of known *B. napus* pre-miRNAs were identified. The miRNA sequences and the number of reads in 24 libraries were listed in Table 3.

**Table 3:** Identification of novel miRNAs on the other arm of known pre-miRNAs in *Brassica napus*

bna-miRNA name	mature miRNA sequence	Length	Read count
bna-miR156f-3p	GCUCACUGCUCUUUCUGUCAGA	22	749
bna-miR156e-3p	UGCUCACCUCUCUUUCUGUCAGU	23	344
bna-miR160a-3p	GCGUAUGAGGAGCCAUGCAUA	21	32
bna-miR160c-3p	GCGUACAGAGUAGUCAAGCAUG	22	24
bna-miR160d-3p	GCGUACAGAGUAGUCAAGCAUG	22	24
bna-miR161-3p	GUCACUUUCAAUGCGUUGAUC	21	7
bna-miR164b/c/d-3p	CACGUGUUCUACUACUCCAAC	21	21
bna-miR166d-5p	GGACUGUUGUCUGGCUCGAGG	21	135
bna-miR166e-5p	GGAAUGUUGUCUGGCACGAGG	21	10
bna-miR166f-5p	GGAAUGUUGUCUGGAUCGAGG	21	202
bna-miR167a/b-3p	GAUCAUGUUCGCAGUUUCACC	21	750
bna-miR167a/b-3p	GAUCAUGUUCGCAGUUUCACC	21	750
bna-miR168a-3p	CCCGCCUUGUAUCAAGUGAAU	21	104
bna-miR171a/b/c-5p	AGAUAUUAGUGCGGUUCAAUC	21	51
bna-miR171d-5p	AGAUAUUGGUGCGGUUCAAUC	21	12
bna-miR172a-5p	GCAGCACCAUCAAGAUUCAC	20	48
bna-miR172b-5p	GCAGCAUCAUUAAGAUUCACA	21	3
bna-miR172c-5p	GCAGCAUCAUCAAGAUUCACA	21	9
bna-miR172d-5p	GCAUCAUCAUCAAGAUUCAGA	21	218
bna-miR2111d-3p	AUCCUCGGGAUACGGAUUACC	21	25
bna-miR390b-3p	CGCUGUCCAUCCUGAGUUUCA	21	1109
bna-miR390c-3p	CGCUAUCCAUCCUGAGUUCC	20	19
bna-miR395a/b/c-5p	GUUCCUCUGAGCACUUCAUUG	21	61
bna-miR395d/f-5p	GUUCCCUUUAACGCUUCAUUG	21	13
bna-miR399b-5p	GGGCAAGAUCUCUAUUGGCAGG	22	12
bna-miR403-5p	UGUUUUGUGCGUGAAUCUAAUU	22	287
bna-miR824-3p	CCUUCUCAUCGAUGGUCUAGA	21	1640

Read count, the total read count of all the small RNA libraries

### Identification of new conserved miRNA families and new miRNA members

To identify new conserved miRNAs in *B. napus*, all mapped small RNAs were mapped to known plant miRNAs in miRBase and *B. napus* genome sequences. If the small RNAs can match known plant miRNAs with no more than three mismatches and can exactly map to *B. napus* genome sequences, then these small RNAs were initially classified as candidate new conserved miRNAs. Five criteria described in the Materials and Methods were used to strictly screen the candidate conserved miRNAs. As a result, 44 miRNAs (22 pairs of miRNAs) belonging to 15 miRNA families were identified (Table 4). Among them, bna-miR159b was a new miRNA member of bna-miR159 family. The rest of the 36 miRNAs (14 pairs of miRNAs) have not been previously reported as bna-miRNAs in miRBase; they show high sequence similarity to some of the known plant miRNAs. The bna-miR158a.1 and bna-miR158a.2 were identified for bna-miR158a member. The two pairs of bna-miR158a shared the same mature sequences. Their precursor sequences were highly similar with each other, and these sequences were from different loci of the *B. napus* genome. These two pairs of miRNAs were called sub-members. This type of sub-member was also observed for bna-miR159b and bna-miR408a. Four sub-members (bna-miR159b.1, bna-miR159b.2, bna-miR159b.3, and bna-miR159b.4) were identified for bna-miR159b, and two sub-members (bna-miR408a.1 and bna-miR408a.2) were identified for bna-miR408a. This phenomenon suggests that some *MIRNA* genes might be produced through a replication event from one origin to another one, which results in more copies of the miRNA group. Two members were identified for bna-miR319 and bna-miR398 families. Except the above mentioned five miRNA families, the rest of 10 miRNA families had only one miRNA member (Table 4). The secondary structures of these new conserved miRNAs were shown in Additional file 2 (Fig. S1).

**Table 4:** Identification of new conserved miRNA families in *Brassica napus*

bna-miRNA	Sequence	Len	Read	pre-position
bna-miR158a.1-5p	CUUUGUCUAUCGUUUGGAAAAG	22	3884	chrA08:2748114..2748220
bna-miR158a.1-3p	UUUCCAAAUGUAGACAAAGCA	21	32,292	
bna-miR158a.2-5p	CUUUGUCUAUCGUUUGGAAAAG	22	3884	chrC08:3581242..3581348
bna-miR158a.2-3p	UUUCCAAAUGUAGACAAAGCA	21	32,292	
bna-miR159b.1-5p	AGCUGCUAAGCUAUGGAUCCC	21	258	chrA02:9865184..9865001
bna-miR159b.1-3p	UUUGGAUUGAAGGGAGCUCUA	21	46,073	
bna-miR159b.2-5p	AGCUGCUAAGCUAUGGAUCCC	21	258	chrA07_random:1944377..1944191
bna-miR159b.2-3p	UUUGGAUUGAAGGGAGCUCUA	21	46,073	
bna-miR159b.3-5p	AGCUGCUAAGCUAUGGAUCCC	21	258	chrC02:19215807..19215624
bna-miR159b.3-3p	UUUGGAUUGAAGGGAGCUCUA	21	46,073	
bna-miR159b.4-5p	AGCUGCUAAGCUAUGGAUCCC	21	258	chrC06:33954934..33954749
bna-miR159b.4-3p	UUUGGAUUGAAGGGAGCUCUA	21	46,073	
bna-miR319a-5p	AGAGCUUCCUUGAGUCCAUUC	21	27	chrC01:10651723.. 10,651,921
bna-miR319a-3p	UUGGACUGAAGGGAGCUCCCU	21	4848	
bna-miR319b-5p	GGAGAUUCUUUCAGUCCAGUC	21	4	chrC04:46407584.. 46,407,846
bna-miR319b-3p	UUGGACUGAAGGGAGCUCCUU	21	27,901	
bna-miR391-5p	UUCGCAGGAGAGAUAGCGCCA	21	110	chrA10:10707678..10707812
bna-miR391-3p	ACGGUAUCUCUCCUACGUAGC	21	237	
bna-miR398a-5p	GGGUCGACAUGAGAACACAUG	21	141	chrA03:2288822..2288945
bna-miR398a-3p	UGUGUUCUCAGGUCACCCCUG	21	9870	
bna-miR398b-5p	GGAGUGUCAUGAGAACACGGA	21	25	chrC02:37793584..37793689
bna-miR398b-3p	UGUGUUCUCAGGUCACCCCUU	21	145	
bna-miR400-5p	UAUGAGAGUAUUAUAAGUCAC	22	78	chrAnn_random:40582790..40582930
bna-miR400-3p	GACUUAUAAUGAUCUCAUGAA	22	237	
bna-miR408a.1-5p	GGGAGCCAGGGAAGAGGCAGU	22	1232	chrA05:478954..479121
bna-miR408a.1-3p	UGCUUGUUCCCUGUCUCUCUC	22	1002	
bna-miR408a.2-5p	GGGAGCCAGGGAAGAGGCAGU	22	1232	chrCnn_random:8448205..8448064
bna-miR408a.2-3p	UGCUUGUUCCCUGUCUCUCUC	22	1002	
bna-miR9554-5p	GAAUGAUACUUGGAUAUAAUC	21	5	chrA06:19718101..19718250
bna-miR9554-3p	UCAUAUCCAAGUAUCAUUCCU	21	81	
bna-miR9558-5p	AGAGAUGUCUGGCUUGCAACA	21	3	chrC03_random:1702602..1702746
bna-miR9558-3p	UUGCAAGCCAGACAUUUCCUUU	22	8	
bna-miR9559-5p	UUUGGAUUUUGGUCAUUGUUG	21	5	chrAnn_random:36404086.. 36,404,194
bna-miR9559-3p	ACAAUGAACGAAAUCCAAAUC	21	3	
bna-miR9560a-5p	ACAGGUGGUGGAACAAAUAUGAGU	25	30	chrA06:19552830..19552965
bna-miR9560a-3p	UCAUAUUAGUUCUACCUCCUGCUG	25	2	
bna-miR9562-5p	ACUAUGCAAUUGUGAACAAAC	21	4	chrA02_random:1408210..1408358
bna-miR9562-3p	UUAUUCACAACUGCAUAAUUC	21	3	
bna-miR9563a-5p	ACCCGUCUCUUAACUUUUAAC	22	15	chrAnn_random:9932700..9932850
bna-miR9563a-3p	UAAAAGUUAAGAGACAAGUUA	22	17	
bna-miR9568-5p	UGCGGAUAUCUUAGGAUGAGGU	22	13	chrA03:13274664..13274813
bna-miR9568-3p	UCAUCGUAAGAGAUCUGCAUU	21	2	
bna-miR9569-5p	UGAGUUAUCAUUGGUCUUGUG	21	1198	chrAnn_random:21855323..21855514
bna-miR9569-3p	ACACAGGAACAAUACUAACUCAUU	24	3509	

*Len* length of mature miRNA, *pre-position* the miRNA precursor sequences in chromosomes of *Brassica napus*, *Read* the total read count of all the small RNA libraries

### Expression profiling of differentially expressed miRNAs in sterile and fertile lines

**Fig. 2:**
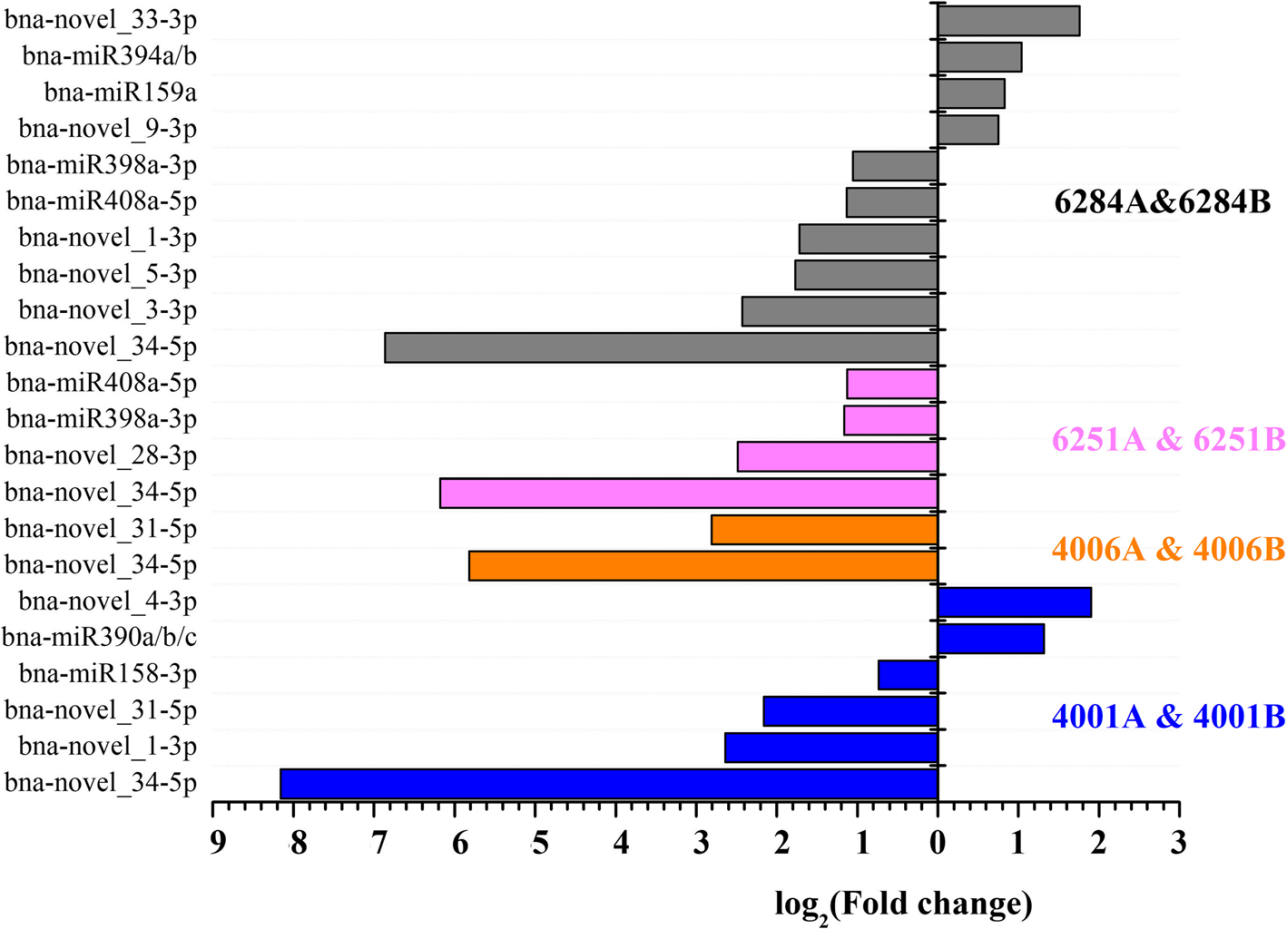
Relative expression analysis of miRNAs in the flower buds of the A line and B line of RGMS and DGMS in *Brassica napus* by high-throughput sequencing*.* Six miRNAs (bule columns) showed significant expression differences between ‘4001A’ and ‘4001B’ flower buds. Two miRNAs (orange columns) showed significant expression differences between ‘4006A’ and ‘4006B’ flower buds. Four miRNAs (red columns) showed significant expression differences between ‘6251A’ and ‘6251B’ flower buds. Ten miRNAs (gray columns) showed significant expression differences between ‘6284A’ and ‘6284B’ flower buds. The Y-axis represents the differentially expressed miRNAs. The X-axis represents the log_2_^(FoldChange)^. Left indicates the miRNAs enriched in B line flower buds. Right indicates the miRNAs enriched in A line flower buds

**Fig. 3:**
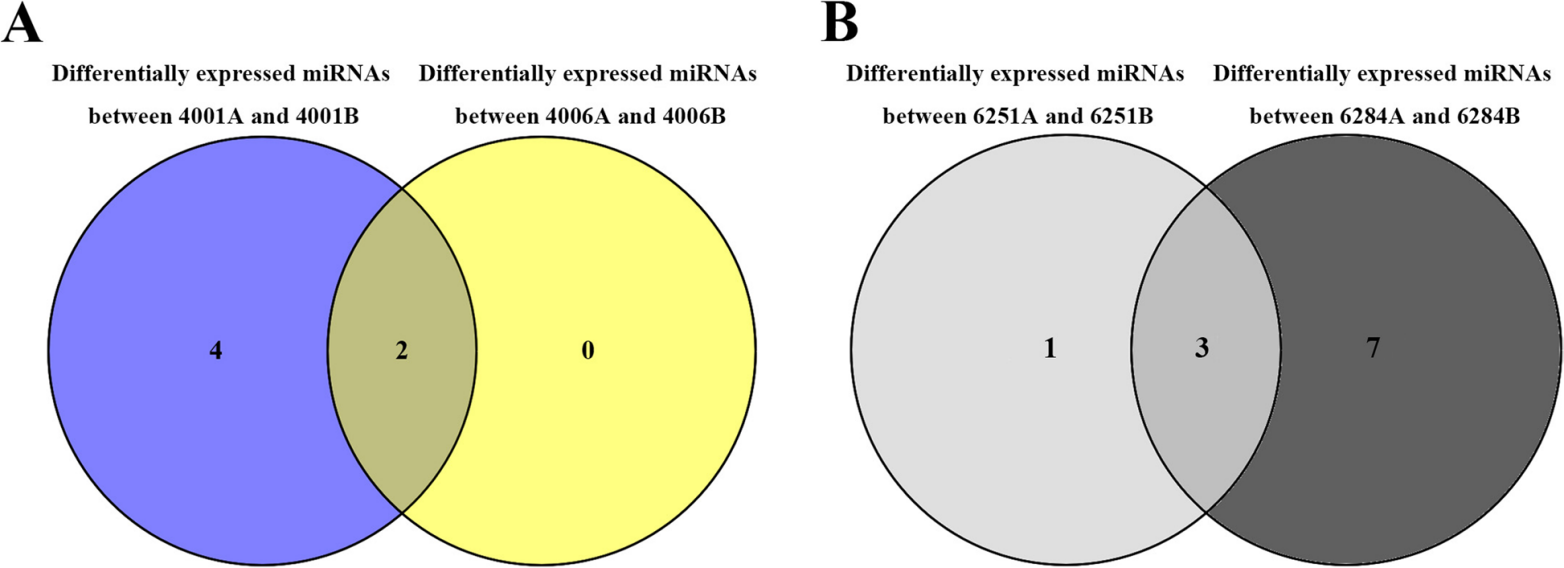
Venn diagram showing overlaps of significantly differential expressed miRNAs between different genic male sterility (GMS) lines of *Brassica napus*. **A** Two differentially expressed miRNAs were shared between 4001AB and 4006AB lines. **B** Three differentially expressed miRNAs were shared between 6251AB and 6284AB lines

The normalized expression levels of miRNAs were used for identifying differentially expressed miRNAs between the sterile line and the corresponding fertile line, such as “4001A” and “4001B”, “4006A” and “4006B”, “6251A” and “6251B”, and “6284A” and “6284B”. The known, identified conserved and novel miRNAs were followed to differential expression analysis criteria (qvalue < 0.01 and |log2 (fold change)| > 0.73). As a result, 6, 2, 4, and 10 differentially expressed miRNAs were obtained between the flower buds of “4001A” and “4001B”, “4006A” and “4006B”, “6251A” and “6251B”, and “6284A” and “6284B”, respectively (Fig. 2, Table 5). To further explore the miRNAs involved in the two DGMS lines, a Venn diagram analysis was conducted. The results indicated that two differentially expressed miRNAs (bna-novel_34-5p and bna-novel_31-5p) were shared between the DGMS lines “4001AB” and “4006AB” (Fig. 3A). In addition, three differentially expressed miRNAs (bna-novel_34-5p, bna-miR408a-5p, and bna-miR398a-3p) were shared between the RGMS lines “6251AB” and “6284AB” (Fig. 3B). The novel miRNA “bna-novel_34-5p” was the only miRNA that was simultaneously differentially expressed in the DGMS and RGMS lines.

**Table 5:** Differentially expressed miRNAs in “4001AB”, “4006AB”, “6251AB” and “6284AB” libraries and their candidate targets by sRNA sequencing and transcriptome analysis in *Brassica napus*

Sample	miR_name	Read (A)	Read (B)	log_2_^(FoldChange)^	Candidate targets
4001	bna-novel_4-3p	8.2	0.4	1.90	BnaC01g31810D,BnaA07g24350D,BnaC02g41520D,BnaA08g22150D,BnaA04g13020D,BnaA07g18960D,BnaA09g38810D,BnaC03g26470D,BnaC02g03200D,BnaA04g00990D,BnaA03g41600D,BnaA05g07140D,BnaA08g19190D
	bna-miR390a/b/c	26.8	11.1	1.32	BnaC03g02760D,BnaC01g03570D,BnaA05g28390D,BnaC03g53360D,BnaC01g22410D
	bna-novel_31-5p	10.7	55.2	−2.16	BnaC01g21190D,BnaA01g17940D,BnaC03g13660D,BnaA03g10950D,BnaA09g00170D,BnaA10g02710D,BnaC01g22250D,BnaA03g38770D,BnaA02g29970D,BnaA02g18470D
	bna-novel_34-5p	0.6	624.0	−8.16	BnaA09g14240D,BnaA05g30840D,BnaA01g24360D,BnaA07g34690D,BnaA03g14400D,BnaA05g09000D,BnaC01g09790D,BnaA08g26410D
	bna-novel_1-3p	0.1	8.1	−2.64	–
	bna-miR158-3p	554.7	935.8	−0.74	BnaA05g08940D,BnaA04g29200D
4006	bna-novel_34-5p	0.2	125.1	−5.82	BnaA05g29360D,BnaA09g14240D,BnaA05g30840D,BnaA01g24360D,BnaA07g34690D,BnaC03g41430D,BnaA03g35600D
	bna-novel_31-5p	4.7	53.3	−2.81	BnaC01g21190D,BnaC03g13660D,BnaA09g19450D,BnaA05g09930D
6251	bna-novel_28-3p	5.2	38.3	−2.48	BnaA09g36810D,BnaA10g16750D
	bna-miR408-5p	23.8	60.6	−1.15	BnaA02g05230D,BnaA10g18650D,BnaA08g27620D,BnaAnng21260D,BnaA10g02870D,BnaA08g00080D
	bna-miR398a-3p	0	5.1	−2.10	BnaA05g02320D,BnaA06g14440D,BnaA08g08840D
	bna-novel_34-5p	23.2	57.5	−1.11	BnaA02g05230D,BnaA10g18650D,BnaC01g01790D,BnaA10g10960D,BnaAnng21260D,BnaA10g14890D,BnaA08g27620D,BnaA07g15260D,BnaAnng36200D,BnaA10g15730D,BnaA02g11840D,BnaA10g25360D,BnaA10g02870D,BnaA08g00080D,BnaA10g17820D,BnaA09g25870D
6284	bna-miR394a/b	195.2	87.5	1.04	BnaA05g16640D,BnaA01g24160D,BnaA05g11890D,BnaC02g17150D,BnaAnng09250D,BnaA08g10740D,BnaA07g36430D,BnaC02g29160D,BnaA06g08380D,BnaC01g07190D,BnaA05g00820D,BnaA05g13120D,BnaC01g39490D,BnaC01g16400D,BnaA02g34270D,BnaC02g43190D,BnaA01g33370D,BnaC01g39860D,BnaA08g27810D
	bna-novel_33-3p	30.2	8.4	1.76	BnaA02g26940D,BnaA09g44810D,BnaA06g12040D,BnaA06g14250D,BnaA02g23840D,BnaA10g26020D,BnaA03g12030D,BnaA09g06400D,BnaC01g11370D,BnaA10g03030D,BnaA03g05700D,BnaA10g26690D,BnaA05g01020D,BnaA02g00710D
	bna-miR159a	2320.1	1295.7	0.82	BnaAnng27960D,BnaA04g18810D,BnaA03g15690D,BnaA07g18670D,BnaA07g12970D,BnaA03g22590D,BnaA07g25350D,BnaA06g20460D,BnaA01g18450D,BnaC01g19500D,BnaA01g16350D,BnaA09g27090D,BnaA06g13170D,BnaA09g42230D,BnaA09g30160D,BnaAnng05670D,BnaA06g18020D,BnaA08g20300D,BnaA02g05410D,BnaAnng21510D,BnaA09g08360D,BnaAnng14630D,BnaA09g02220D,BnaA04g25320D,BnaA09g10390D,BnaA02g30030D,BnaA09g44380D,BnaC01g19800D,BnaA01g35420D,BnaA08g27930D
	bna-novel_9-3p	114.4	68.3	0.75	BnaA03g33680D,BnaA08g01260D,BnaA01g05980D,BnaAnng27960D,BnaA03g22590D,BnaA07g12970D,BnaA04g09220D,BnaA07g12970D,BnaA02g05410D,BnaA09g55500D,BnaAnng13060D,BnaA01g23170D,BnaA09g13960D,BnaA09g47880D,BnaA04g01370D,BnaA08g17490D,BnaC02g08160D,BnaA02g33550D,BnaC02g42310D,BnaA10g14420D,BnaA05g16460D,BnaA03g42760D
	bna-miR398a-3p	436.2	972.6	−1.06	BnaA08g19040D,BnaC02g28060D,BnaC01g27860D,BnaA10g26450D,BnaC03g13330D,BnaA03g10640D,BnaC02g12070D,BnaC01g18280D,BnaA01g15390D,BnaC02g42090D,BnaAnng31090D,BnaC01g43200D,BnaA05g30940D,BnaA01g29240D,BnaC01g36670D
	bna-novel_3-3p	3.1	23.4	−2.43	BnaA05g24640D,BnaA09g38650D,BnaA06g39700D,BnaA09g39360D,BnaA09g54170D,BnaA06g10130D,BnaA10g14600D,BnaC01g22370D,BnaA10g24950D,BnaA10g23300D,BnaA04g14120D,BnaA09g41640D,BnaC01g09540D
	bna-novel_5-3p	1.5	13.5	−1.77	BnaA05g24640D,BnaA06g39700D,BnaC02g14840D,BnaA06g10130D,BnaA09g38650D,BnaC01g22370D,BnaA09g39360D,BnaA10g04390D,BnaA09g54170D,BnaA10g14600D,BnaA01g28170D,BnaA06g28940D,BnaA10g23460D,BnaA06g07690D,BnaA10g24950D,BnaA09g01710D,BnaA10g16750D,BnaA10g23300D,BnaC01g09540D
	bna-miR408-5p	41.3	94.4	−1.13	BnaA03g22490D,BnaA10g18290D,BnaA07g26450D,BnaA06g37530D,BnaC02g29240D,BnaA05g29880D,BnaA10g21900D,BnaC01g16210D,BnaA02g09770D,BnaA10g23140D,BnaA02g34310D,BnaC02g43230D,BnaA01g08900D,BnaA06g10230D,BnaA10g15210D,BnaA05g14430D,BnaC02g46010D,BnaA03g04360D,BnaA04g08040D,BnaAnng32010D,BnaA03g30060D
	bna-novel_1-3p	0.9	7.5	−1.72	BnaA02g30100D,BnaC02g38520D,BnaA10g21900D,BnaAnng29380D
	bna-novel_34-5p	1.0	456.9	−6.87	BnaA02g05300D,BnaA05g27100D,BnaA07g28780D,BnaA06g28070D,BnaA09g14240D,BnaA05g30840D,BnaA08g29690D,BnaC03g48340D,BnaA04g22150D,BnaC01g31340D,BnaA01g24360D,BnaA03g23950D,BnaC03g53270D,BnaA06g01440D,BnaA02g14960D,BnaA09g03120D,BnaA09g31030D,BnaC02g03920D,BnaA07g34690D,BnaA03g35600D,BnaA04g02140D,BnaC02g14950D,BnaA03g10460D,BnaAnng21410D

*Read (A)* Read count in sterile lines (TPM), *Read (B)* Read count in fertile lines (TPM), *log*_*2*_^*(FoldChange)*^ log_2_^(A/B)^

**Fig. 4:**
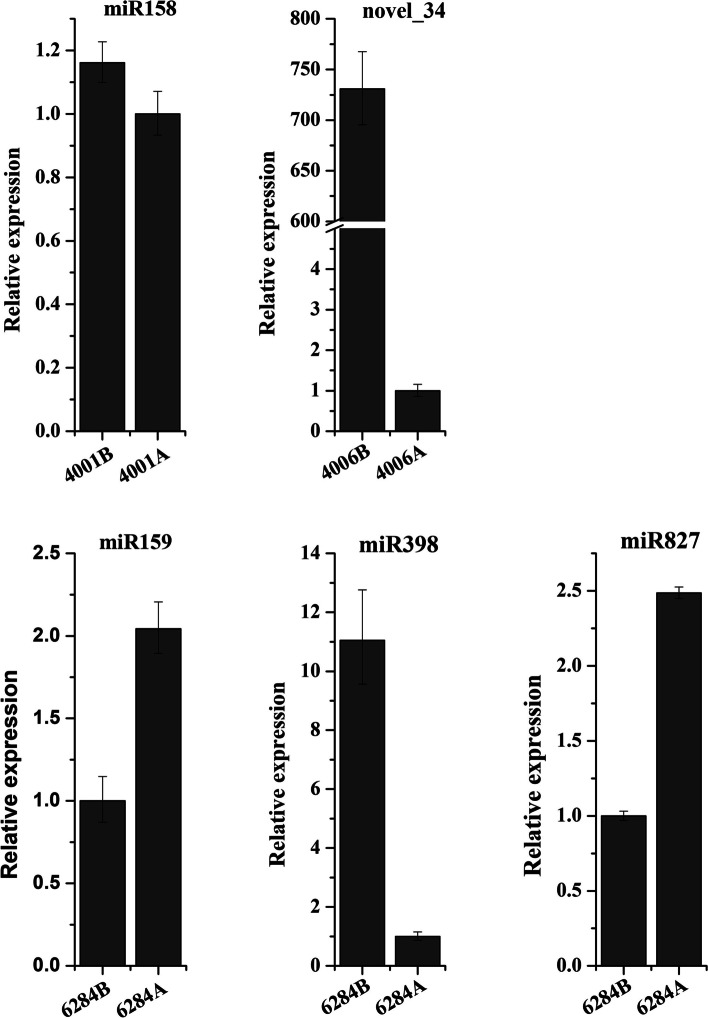
The qRT-PCR analysis of differentially expressed miRNAs between the flower buds of A lines and B lines. The flower buds used for qRT-PCR analysis were collected from corresponding A lines or B lines

qRT-PCR was conducted to verify the expression profiles of these differentially expressed miRNAs in deep sequencing. Five differentially expressed miRNAs were chosen for qRT-PCR analysis. The results were consistent with those of deep sequencing. In qRT-PCR, miR158 was up-regulated in “4001B” flower buds compared with that in “4001A”. Novel_34 was greatly up-regulated in “4006B” flower buds (730-fold) compared with that in “4006A”. MiR159 and miR827 were both up-regulated in “6284A” flower buds compared with that in “6284B”. miR398 was up-regulated in “6284B” flower buds compared with that in “6284A” (Fig. 4).

### Target prediction and identification of differentially expressed miRNAs in sterile and fertile lines

A plant small RNA target analysis server (psRNATarget)-based analysis was performed to predict miRNA target genes with default parameters and a maximum expectation value of 3.5 (https://www.zhaolab.org/psRNATarget/). A total of 560 transcripts were predicted to be targets of the 15 miRNAs (Additional Table S3). In addition, transcriptome sequencing was conducted using the same samples as sRNA sequencing (unpublished data). The differentially expressed and up-regulated mRNAs were predicted as the candidate targets for the differentially expressed and down-regulated miRNAs. At the same time, the differentially expressed and down-regulated mRNAs were predicted as the candidate targets for the differentially expressed and up-regulated miRNAs. As shown in Table 5. Thirty-eight candidate target genes were predicted for the six differentially expressed miRNAs between “4001A” and “4001B”. Eleven candidate genes were predicted for the two differentially expressed miRNAs between “4006A” and “4006B”. Twenty-seven candidate genes were predicted for the four differentially expressed miRNAs between “6251A” and “6251B”. One hundred and eighty-one candidate genes were predicted for the ten differentially expressed miRNAs between “6284A” and “6284B”.

**Fig. 5:**
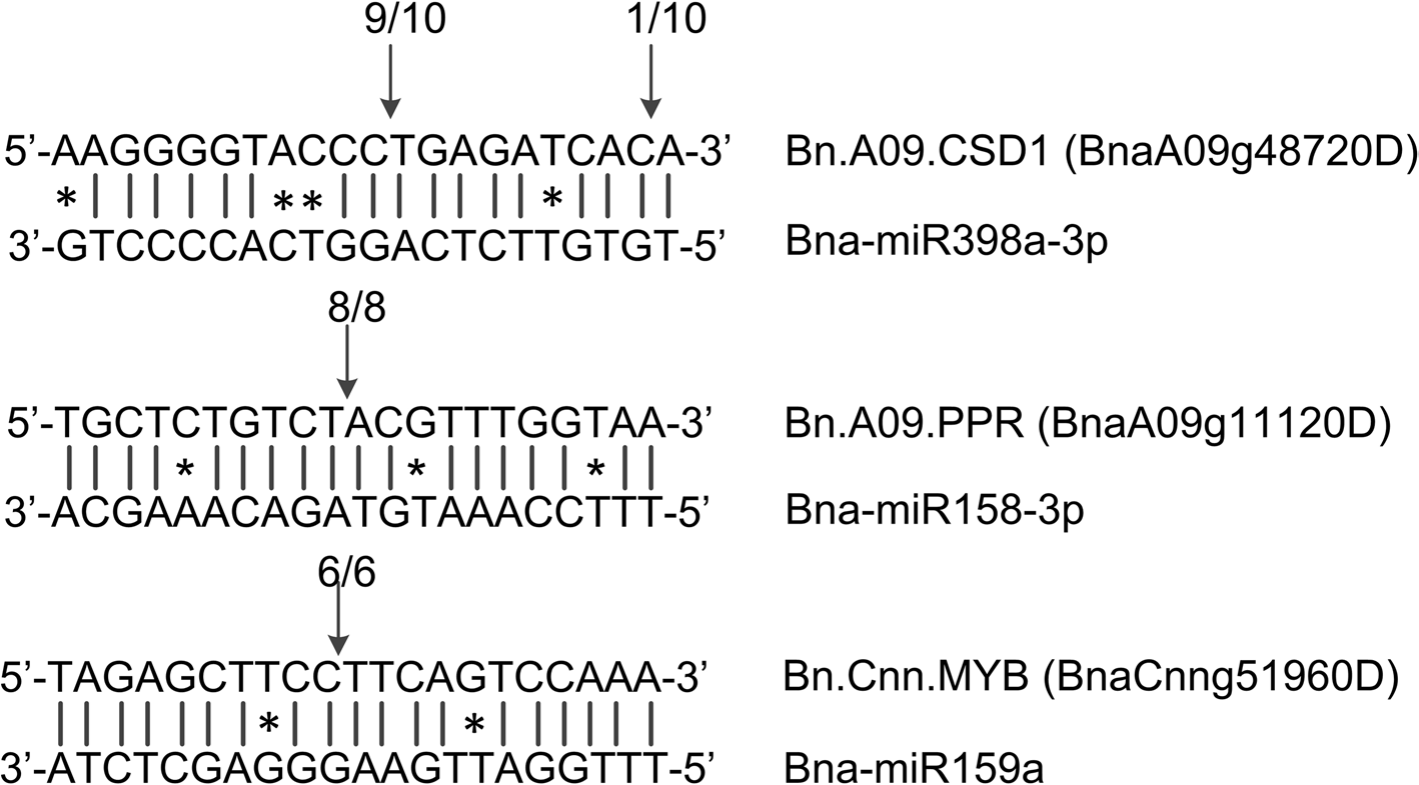
Identification of miRNA target genes in *B. napus* by 5′ modified RACE. The arrows show the cleavage sites of target mRNA. Watson-Crick pairing and mismatches are indicated by vertical dashes and asterisks

To further demonstrate the potential target genes, 5′ modified RACE was performed using mixed samples from flower buds of the fertile lines (“6284B” and “4001B”). Three target genes were validated using 5′ modified RACE (Fig. 5). *Bn.A09.CSD1* (*BnaA09g48720D*) was cleaved by bna-miR398a-3p. *Bn.A09.PPR* (*BnaA09g11120D*) was cleaved by bna-miR158-3p. *Bn.Cnn.MYB* (*BnaCnng51960D*) was cleaved by bna-miR159a.

### Overexpression of bna-miR159a affected seeds and siliques development in *Arabidopsis*

**Fig. 6:**
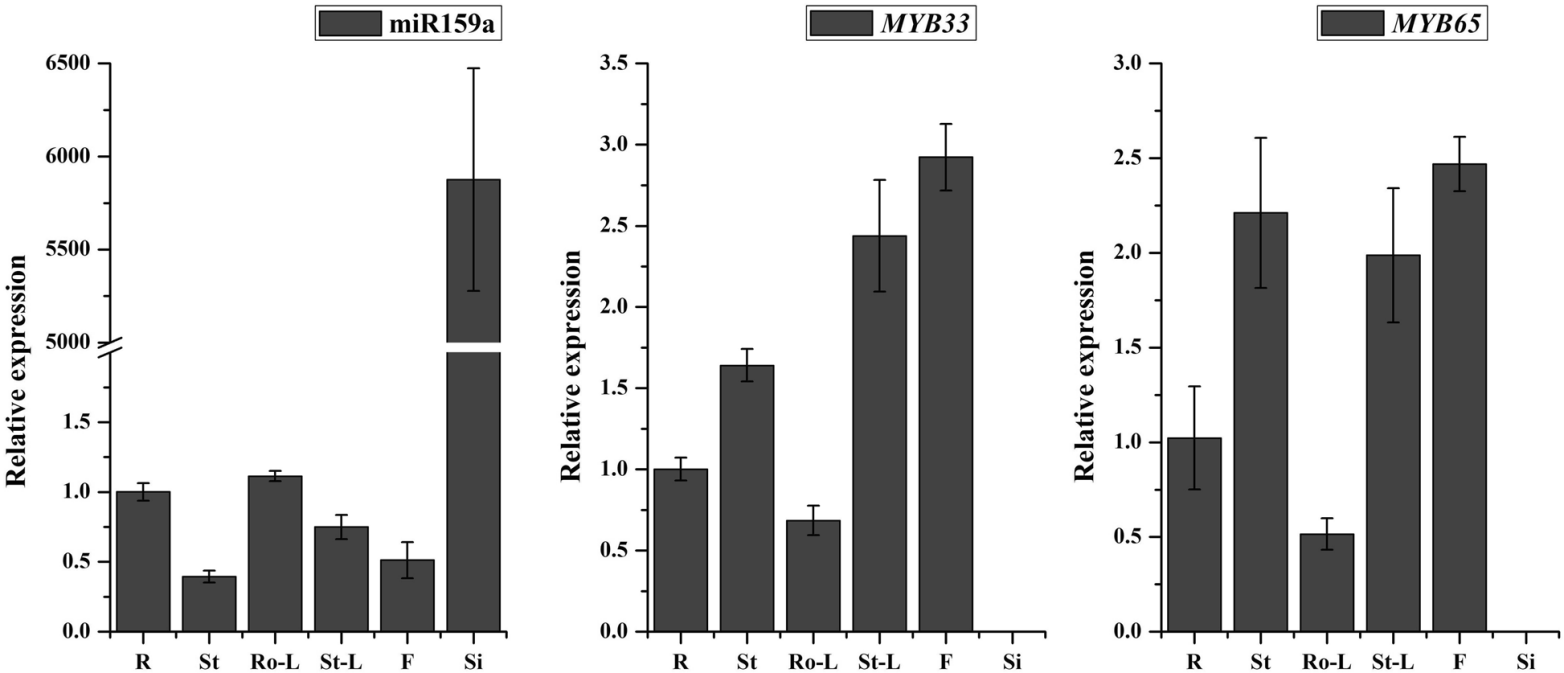
The relative expression levels of miR159 and its targets in different tissues from wild type plants of *Arabidopsis*. R, root; St, stem; Ro-L, rosette leaf; St-L, stem leaf; F, flower; Si, silique

**Fig. 7:**
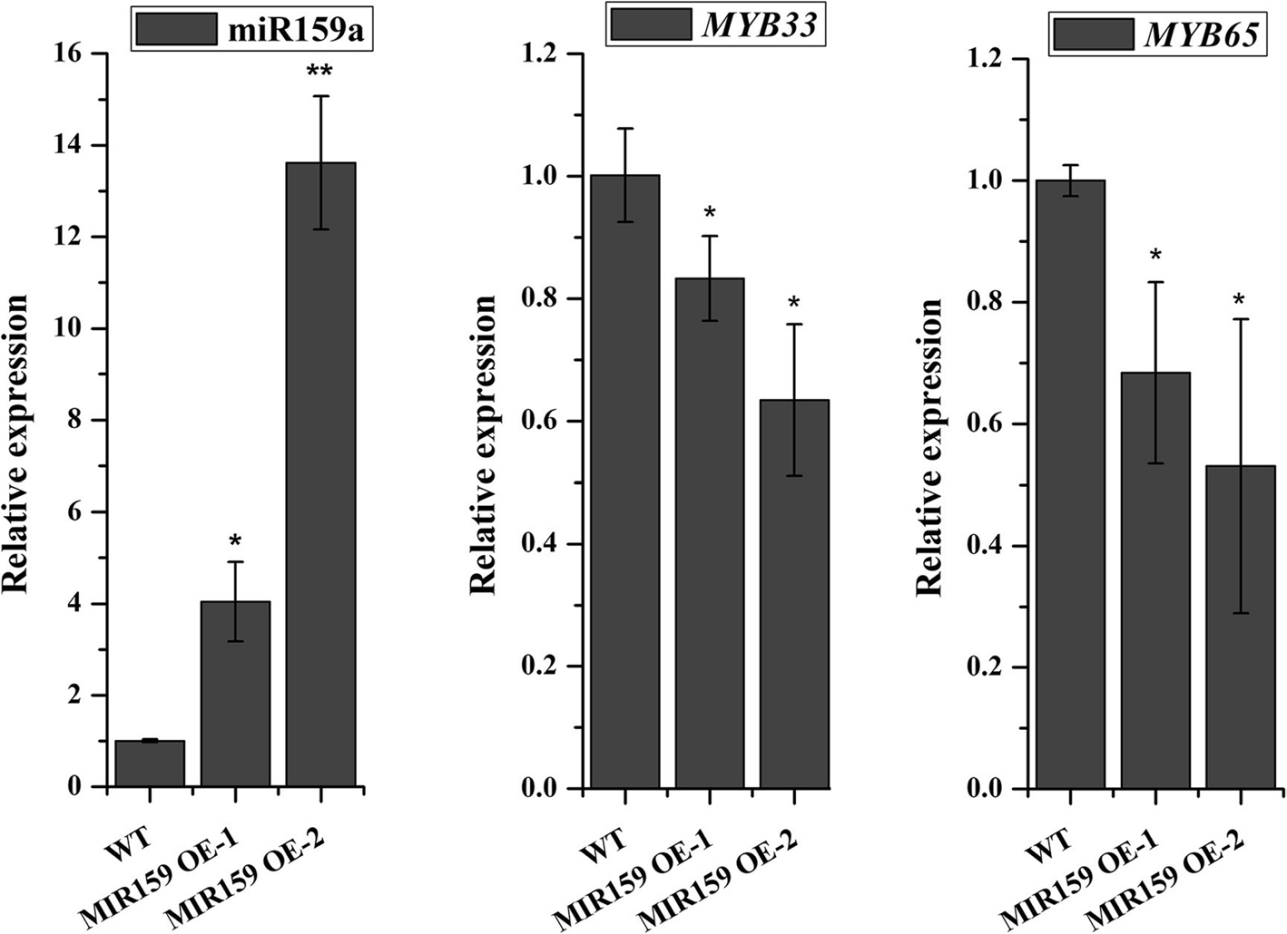
The relative expression levels of miR159 and its targets in stem leaf of MIR159OE transgenic lines of *Arabidopsis*. In the T_2_ transgenic plants, stem leaf from line 1 of MIR159OE-1 and mixed stem leaf from line 3 and line 4 of MIR159OE-2 were used for qRT-PCR analysis

Among all the differentially expressed miRNAs in the two DGMS and RGMS lines, bna-miR159a had the highest expression level. To reveal miR159 potential function, two constructs containing pre-miR159a-C6 and pre-miR159a-A7 were transformed to *Arabidopsis,* and corresponding MIR159OE-1 and MIR159OE-2 transgenic plants were obtained. Five and four lines were obtained for MIR159OE-1 and MIR159OE-2 in T_1_, respectively. In wild-type plants of *Arabidopsis*, the transcript of mature miR159a and its targets (*AtMYB33* and *AtMYB65*) were detected in root, stem, rosette leaf, stem leaf, flower, and silique through qRT-PCR. The expression level of mature miR159a was the highest in silique (5875-fold), relatively lower in stem, stem leaf, and flower compared with that in root. The expression levels of *AtMYB33* and *AtMYB65* were very low and almost undetectable in silique, whereas they were relatively high in stem, stem leaf, and flower compared with that in the root (Fig. 6). In T_2_ transgenic plants, the transcripts of miR159a and its targets were detected in stem leaf from line 1 of MIR159OE-1 and mixed stem leaf from line 3 and line 4 of MIR159OE-2. The results indicated mature miR159a was overexpressed in MIR159OE-1 (4.04-fold) and MIR159OE-2 (13.6-fold) compared with that in WT. Meanwhile, the transcripts of *AtMYB33* and *AtMYB65* were suppressed in MIR159OE-1 and MIR159OE-2, especially in MIR159OE-2, compared with that in WT (Fig. 7).

**Fig. 8:**
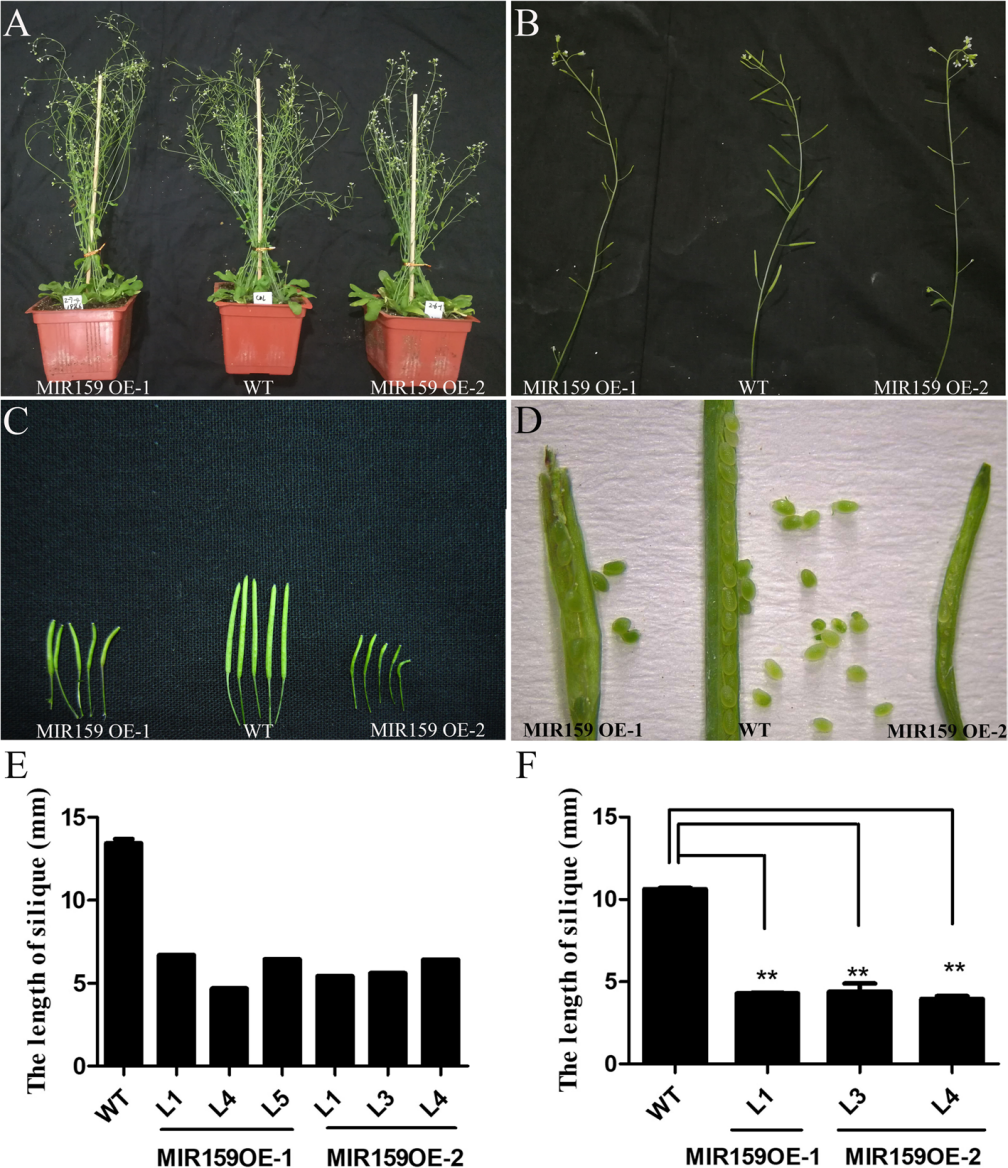
The phenotypes of MIR159OE transgenic lines. The T_2_ transgenic plants (A) and siliques (B-D) of MIR159OE-1 and MIR159OE-2 were observed. The length of siliques was measured in T_1_ (E) and T_2_ (F) transgenic plants of MIR159OE-1 and MIR159OE-2. L1, L3, L4, L5 indicate line 1, line 3, line 4, and line 5. More than 30 siliques for each plant were measured. Data represent means of three replicates ± SD. *, *P < 0.05*, **, *P < 0.01*. Student’s *t*-test

The morphological characters of MIR159OE-1, MIR159OE-2, and WT were observed along with their development processes, especially in the flowering and fruiting periods. No significant difference was observed between transgenic and WT plants in the vegetative growth phase. However, during the reproductive growth period, in the T_1_ and T_2_ transgenic plants of MIR159OE-1 and MIR159OE-2, the seed setting rate decreased, and siliques became shorter compared with that in the WT (Fig. 8). The length of siliques from WT, MIR159OE-1, and MIR159OE-2 transgenic plants were measured. In T_1_, the silique length of WT was approximately 13.4 mm, while in the MIR159OE-1 transgenic plants, the silique lengths of line 1, line 4, and line 5 were 6.7 mm, 4.6 mm and 6.4 mm, respectively. And the silique lengths of line 1, line 3, and line 4 of MIR159OE-2 were 5.4 mm, 5.6 mm, and 6.4 mm, respectively (Fig. 8E). In T_2_, the silique length of WT was approximately 10.6 mm, while in the MIR159OE-1 plants, the silique length of line 1 was 4.3 mm. The silique lengths of line 3 and line 4 of MIR159OE-2 were 4.4 mm and 4.0 mm, respectively (Fig. 8F). These results indicated that overexpression of *MIR159* resulted in significantly shorter siliques and reduced seed setting rate.

## Discussion

MiRNAs, as the key post-transcriptional regulators, participate in various biological processes in plant. Recently, an increasing number of studies showed that plant miRNAs were also involved in pollen and fertility development. In 2014, Jiang et al. identified 54 new conserved miRNAs and 25 pairs of novel miRNA/miRNA* in a GMS system of *B. campestris* ssp. *chinensis*. Eighteen differentially expressed miRNAs with over two fold change between flower buds of male sterile and fertile lines were identified; they might be involved in the pollen development process [25]. In 2017, Ma et al. verified that the overexpression of *MIR158* caused pollen abortion and reduced pollen vitality, which were caused by the degradation of pollen content from the binuclear microspore stage [26]. Dong et al. identified 85 known miRNAs and 120 novel miRNAs, which were expressed during rapeseed anther development in a novel recessive GMS system “CN12AB.” Moreover, 19 and 18 known miRNAs were found to be differentially expressed in 0.5–1.0 mm buds and in 2.5–3.0 mm buds between CN12A and CN12B, respectively. Among these, 14 miRNAs were up-regulated, and 23 miRNAs were down-regulated expressed in CN12A compared with those in CN12B [27]. In this study, to identify miRNAs and their targets involved in pollen development and GMS occurrence in rapeseed, 24 small RNA libraries and transcriptome libraries were constructed and sequenced for the flower buds from the fertile and sterile lines of two RGMS lines (“6251AB” and “6284AB”) and two DGMS lines (“4001AB” and “4006AB”). Based on the sequencing results, fifteen differentially expressed miRNAs with over 1.5-fold change between flower buds of male sterile and fertile lines were identified, including six differentially expressed miRNAs between “4001A” and “4001B”, two differentially expressed miRNAs between “4006A” and “4006B”, four differentially expressed miRNAs between “6251A” and “6251B”, and ten differentially expressed miRNAs between “6284A” and “6284B”. Among them, bna-novel_34-5p was common and differentially expressed between the fertile and sterile lines of “4001AB”, “4006AB”, “6251AB”, and “6284AB”. The results of previous studies and this study verified that miRNAs may play important regulatory roles in rapeseed pollen development and GMS occurrence.

MiR159 is conserved in many plants and is involved in multiple growth and development processes of plants. Allen et al. obtained a *mir159ab* double mutant in *Arabidopsis*, which showed pleiotropic morphological defects, including altered growth habit, curled leaves, small siliques, and small seeds [28]. Millar and Gubler verified that miR159 regulated anther development by regulating the expression of its targets, such as *MYB33* and *MYB65* [29]. Overexpression of miR159 caused the down-regulation of *MYB103* transcripts and earlier degeneration of the tapetum and aberrant pollen formation during anther development [30]. In radish, differential expression level of miR159 during anther development was observed among male sterile and maintainer lines. Increased levels of miR159 transcripts decreased the expression of *MYB101*, thereby inhibiting tapetum development and exine formation [31]. Chen et al. identified 17 differentially expressed miRNAs between long and short siliques of rapeseed, including miR159. Correlation analysis of miR159 and its targets suggested that miR159 repressed cell proliferation to control silique length [15]. Hu et al. found that the overexpression of Bra-*MIR159a* caused pollen abortion and abnormal pollen germination [32]. In this study, the differentially expressed miR159 in “6284A” and “6284B” was chosen to analyze its function during anther development. The bna-miR159 was overexpressed in *Arabidopsis* and resulted in decreased seed setting rate, and shortened siliques, illustrating that miR159 may regulate the fertility and silique development of rapeseed. The results of previous reports and the results of the present study verified that miR159 and its target genes might be involved in the regulatory network of pollen development and male sterility and this module is conserved in plants.

## Conclusion

A large number of miRNAs were identified during pollen development in the two DGMS and two RGMS lines by deep sequencing. These identified miRNAs included 27 novel miRNAs on the other arm of known pre-miRNAs, 44 new conserved miRNAs, and 35 pairs of novel miRNA-3p/miRNA-5p. Among all the identified miRNAs, 15 differentially expressed miRNAs with over 1.5-fold change between flower buds of male sterile and fertile lines were identified, including six differentially expressed miRNAs between “4001A” and “4001B”, two differentially expressed miRNAs between “4006A” and “4006B”, four differentially expressed miRNAs between “6251A” and “6251B”, and ten differentially expressed miRNAs between “6284A” and “6284B”. The qRT-PCR results of 5 differentially expressed miRNAs (miR158, novel_34, miR159, miR827, and miR398) were consistent with deep sequencing results. The association analysis of small RNA and transcriptome sequencing was conducted, and the analysis results indicated that 257 genes were predicted to be the candidate targets of 15 differentially expressed miRNAs. The results of 5′ modified RACE verified that *Bn.A09.CSD1* (*BnaA09g48720D*), *Bn.A09.PPR* (*BnaA09g11120D*), and *Bn.Cnn.MYB* (*BnaCnng51960D*) were cleaved by bna-miR398a-3p, bna-miR158-3p, and bna-miR159a. Additionally, overexpression of bna-miR159 in *Arabidopsis* resulted in decreased seed setting rate, and shortened siliques, illustrating that miR159 may regulate rapeseed fertility and silique development. All the results in our study would provide valuable clues for exploring miRNA-mediated regulatory networks in fertility development of GMS lines in *B. napus*.

## Methods

### Plant materials

“6251AB” and “6284AB” are two recessive genic male sterile (RGMS) lines of *B. napus*. The “6251A” and “6284A” are male sterile lines and the “6251B” and “6284B” are the fertile lines. Their male sterility is controlled by two loci (*BnMs3* and *BnRf*) [14]. The original source of the two RGMS lines was “9012A”, which was identified by Chen et al. [9] and Sun et al. [33]. “4001AB” and “4006AB” are two dominant genic male sterile (DGMS) lines of *B. napus*. The “4001A” and “4006A” are male sterile lines and the “4001B” and “4006B” are the fertile lines. Their male sterility is controlled by three alleles (*Mf*, *Ms*, and *ms*) at the same locus [5]. The original source of the two DGMS lines was “Yi3A”, which was identified by Li et al. [34]. The above RGMS lines and DGMS lines used in this study have been grown for several generations. The progenies of the two RGMS lines are both segregated into sterile and fertile types during reproduction at a ratio of 1:1. While the two DGMS lines are segregated into sterile and fertile types at a ratio of 3:1. These plant materials were planted in the experimental farm of Zhuanghang comprehensive experimental station of Shanghai Academy of Agricultural Sciences. During flowering stage, mixed flower buds were respectively harvested from more than ten plants of the eight lines. The eight lines were “6251A”, “6251B”, “6284A”, “6284B”, “4001A”, “4001B”, “4006A”, and “4006B”. The eight kinds of samples were quickly frozen in liquid nitrogen and stored at − 80 °C. Three independent biological replicates were collected for each kind of sample.

### Small RNA library construction and sequencing

Total RNA for each kind of sample was extracted in three biological replicates using Trizol reagent (Invitrogen, USA). RNA samples with an OD260/OD280 ratio of 2.0 and a total content of more than 2 μg were qualified for small RNA library construction. Then, 24 sequencing libraries (three biological replicates respectively for “6251A”, “6251B”, “6284A”, “6284B”, “4001A”, “4001B”, “4006A”, and “4006B”) were constructed using TruSeqSmall RNA Sample Preparation Kit (Illumina, USA) and then sequenced using Illumina Hiseq 2500/Miseq at Beijing Novogene Bioinformatics Technology Co. Ltd.

### Data analysis

Clean reads were obtained by removing low-quality reads, N-containing fragments, and adapters. Then, the length of 18 to 30 nt clean reads were mapped to the *B. napus* genome sequence (http://brassicadb.agridata.cn/brad/) using Bowtie2 with no mismatches allowed, more details were described in Niu et al. [35]. Unmapped sequences were removed.

The mapped small RNAs reads were aligned to known miRNAs in miRBase22.1. Modified software mirdeep2 [36] were used to predict the potential miRNAs and secondary structures. The ncRNAs includes rRNAs, tRNAs, snRNAs, snoRNAs, and small genome repeat sequences were removed. The rest of sRNA sequences were aligned to *B. napus* NAT-siRNAs in PlantNATsDB to remove NAT-siRNAs. Then miREvo [37] and mirdeep2 [36] were used to predict novel miRNAs in *B. napus*. To reveal the differentially expressed miRNAs, the miRNAs expression was analyzed using the DESeq2 [38]. The online software of Venny 2.1.0 was used to draw Venn diagrams.

### Identification of conserved and novel miRNAs

The candidate sRNAs were mapped to all the known plant miRNA sequences from the miRBase database (http://www.mirbase.org/). The matched sRNAs with no more than three mismatches were considered as candidate conserved miRNAs, while the unmatched sRNAs were considered as candidate novel miRNAs. In addition, Mfold software was used to predict the secondary structures of pre-miRNAs with the flanking sequences of the candidate small RNAs in the genome [39]. Five criteria must be met for identifying conserved and novel miRNAs [40, 41]. The sequences and lengths, read counts, and positions in chromosome were further analyzed for conserved and novel miRNAs.

### qRT-PCR

Total RNA was treated with DNase I (Takara, Japan) to remove residual genomic DNA. For the qRT-PCR analysis of *AtMYB33* and *MYB65*, *AtACTIN2* gene was used as internal control. PrimeScript™ II reverse transcriptase (Takara) and oligo (dT) primers were used for first-strand cDNA synthesis. For the qRT-PCR analysis of mature miRNAs, U6 was used as internal control gene, and the first cDNA was synthetized using a miRNA First Strand cDNA Synthesis kit (Stem-loop Method) (Sangon Biotech). The qRT-PCR reactions were performed in a MyiQ2 qRT-PCR detection system (Bio-Rad, www.bio-rad.com/) using iQ SYBR Green supermix (Bio-Rad) [42]. Each experiment was conducted in three biological replicates, and the same sample was performed in three technical replicates. Relative expression levels of miRNAs and their target genes were quantified by using the 2^-ΔΔCt^ method [43]. All the primers used for qRT-PCR analysis are listed in Additional file 1: Table S4.

### 5′ modified RACE analysis

A mixture of flower buds from the fertile lines (“6284B” and “4001B”) was used for total RNA isolation. The 5′ modified RACE was performed using a FirstChoice™ RLM-RACE Kit (Invitrogen, USA). Total RNA was directly ligated to the 5’RACE oligo. The first-strand cDNA synthesis and the two rounds of PCR reactions were conducted following the manufacturer’s instructions. The PCR products containing the target gene bands were ligated into pGEM-T Easy Vector (Promega, USA) for sequencing [25]. The primers are listed in Additional file 1: Table S4.

### Vector construction and plant transformation

Two precursor sequences of bna-miR159a, which were located in A7 and C6 chromosomes of *B. napus* were designated as pre-miR159a-C6 and pre-miR159a-A7, respectively (Additional file 1: Table S5). The 411 nt and 470 nt genomic fragments containing pre-miR159a-C6 and pre-miR159a-A7 were amplified from *B. napus* using gene specific primers with endonuclease cleavage sites *Sma* I and *Sal* I. Then the fragments were cloned into pCAMBIA1301 binary vector with CaMV 35S promoter. The two vectors were designated as p35S::MIR159a-C6 and p35S::MIR159a-A7, which were introduced into *Agrobacterium tumefaciens* strains GV3101 and further transformed into *Arabidopsis* by floral dip. The inflorescences were dipped in the *Agrobacterium* solution containing sucrose and Silwet-77 for 2 min. The infected plants were cultured for 48 h in the dark environment and then transferred to greenhouse [44]. The seeds were harvested and screened by germination on MS medium containing 25 mg/L hygromycin. The T_1_ and T_2_ hygromycin-resistant seedlings were transplanted and grown in greenhouse. Seeds from each transgenic plant were harvested separately. The corresponding *Arabidopsis* transgenic plants were designated as MIR159OE-1 and MIR159OE-2. The transgenic and wild-type plants were cultivated in the greenhouse under the same environment. The phenotypes of T_1_ and T_2_ transgenic plants were observed and recorded. Their silique length data were collected from more than three plants for each transgenic line. More than 30 siliques were measured for each plant.

## Supplementary Information

**Additional file 1: Table S1.** Identification of known miRNAs. **Table S2.** Precursors of 35 novel miRNAs in *Brassica napus*. **Table S3.** Target genes were predicted by psRNATarget. **Table S4.** The primers were used for qRT-PCR and 5^,^ modified RACE analysis. **Table S5.** The sequences used for constructing MIR159 over-expressed vectors.

**Additional file 2: Figure S1.** The secondary structures of new conserved miRNAs and novel miRNAs identified in *Brassica napus*.

## Data Availability

The raw reads of the 24 sRNA libraries were uploaded to SRA database of NCBI and 24 accession numbers were obtained, including SRX11350295, SRX11350296, SRX11350307, SRX11350312, SRX11350313, SRX11350315, and SRX11350316 (https://dataview.ncbi.nlm.nih.gov/object/PRJNA743414?reviewer=t674c02cj415380e8oldre4s5a). The raw transcriptome data will also be further utilized to excavate differentially expressed mRNAs in the fertile and sterile lines of RGMS and DGMS line.

## Abbreviations

GMS: Genic male sterility
RGMS: Recessive genic male sterility
DGMS: Dominant genic male sterility
CMS: Cytoplasmic male sterility
