# Supplementary material for: Identification of miRNAs and their target genes in genic male sterility lines in Brassica napus by small RNA sequencing
Source: BMC Plant Biol. 2021 Nov 9;21:520. doi: 10.1186/s12870-021-03306-w (PMC8576947; doi:10.1186/s12870-021-03306-w)
Supplement: Supplementary file 1 — Additional file 1: Table S1. Identification of known miRNAs. Table S2. Precursors of 35 novel miRNAs in Brassica napus. Table S3. Target genes were predicted by psRNATarget. Table S4. The primers were used for qRT-PCR and 5, modified RACE analysis. Table S5. The sequences used for constructing MIR159 over-expressed vectors. [file 12870_2021_3306_MOESM1_ESM.docx]

**Table S1 Identification of known miRNAs in *Brassica napus***

| miR_name | Mature sequence (5' - 3') | Len | Read |
| --- | --- | --- | --- |
| bna-miR1140 | ACAGCCUAAACCAAUCGGAGC | 21 | 468 |
| bna-miR156d/e/f-5p | UGACAGAAGAGAGUGAGCAC | 20 | 353 |
| bna-miR156b/c/g | UUGACAGAAGAUAGAGAGCAC | 21 | 304 |
| bna-miR159a | UUUGGAUUGAAGGGAGCUCUA | 21 | 46073 |
| bna-miR160a/b/c/d-5p | UGCCUGGCUCCCUGUAUGCCA | 21 | 409 |
| bna-miR161-5p | UCAAUGCACUGAAAGUGACUA | 21 | 82 |
| bna-miR162a | UCGAUAAACCUGUGCAUCCAG | 21 | 20 |
| bna-miR164a | UGGAGAAGCAGGGCACGUGCA | 21 | 658 |
| bna-miR164b/c/d | UGGAGAAGCAGGGCACGUGCG | 21 | 519 |
| bna-miR166f | UCGGACCAGGCUUCAUCCCCC | 21 | 3875 |
| bna-miR166a/b/c/d/e | UCGGACCAGGCUUCAUUCCCC | 21 | 13671 |
| bna-miR167c | UGAAGCUGCCAGCAUGAUCUA | 21 | 10739 |
| bna-miR167d | UGAAGCUGCCAGCAUGAUCU | 20 | 7569 |
| bna-miR168a | UCGCUUGGUGCAGGUCGGGAA | 21 | 817 |
| bna-miR168b | UCGCUUGGUGCAGGUCGAGAA | 21 | 8 |
| bna-miR169a/b | CAGCCAAGGAUGACUUGCCGA | 21 | 1 |
| bna-miR169n | CAGCCAAGGAUGACUUGCCGG | 21 | 4 |
| bna-miR169m | UGAGCCAAAGAUGACUUGCCG | 21 | 61 |
| bna-miR171f | UGAUUGAGCCGCGCCAAUAUC | 21 | 1778 |
| bna-miR171a/b/c/d/e | UUGAGCCGUGCCAAUAUCACG | 21 | 85 |
| bna-miR172a | AGAAUCUUGAUGAUGCUGCAU | 21 | 488 |
| bna-miR172d | AGAAUCUUGAUGAUGCUGCAG | 21 | 658 |
| bna-miR172b/c | GGAAUCUUGAUGAUGCUGCAU | 21 | 86 |
| bna-miR2111a-3p | GUCCUCGGGAUGCGGAUUACC | 21 | 2 |
| bna-miR2111a/b-5p/d | UAAUCUGCAUCCUGAGGUUUA | 21 | 7 |
| bna-miR2111b-3p | AUCCUCGGGAUACAGAUUACC | 21 | 12 |
| bna-miR390a/b/c-5p | AAGCUCAGGAGGGAUAGCGCC | 21 | 1171 |
| bna-miR390b-3p | CGCUGUCCAUCCUGAGUUUCA | 21 | 1109 |
| bna-miR393-5p | UCCAAAGGGAUCGCAUUGAUC | 21 | 32 |
| bna-miR394a/b | UUGGCAUUCUGUCCACCUCC | 20 | 3164 |
| bna-miR395a/b/c | CUGAAGUGUUUGGGGGAACUC | 21 | 648 |
| bna-miR395d/e/f | CUGAAGUGUUUGGGGGGACUC | 21 | 66 |
| bna-miR396a | UUCCACAGCUUUCUUGAACUU | 21 | 1778 |
| bna-miR399a/b | UGCCAAAGGAGAUUUGCCCGG | 21 | 6 |
| bna-miR403-3p | UUAGAUUCACGCACAAACUCG | 21 | 5956 |
| bna-miR6028 | UGGAGAGUAAGGACAUUCAGA | 21 | 59 |
| bna-miR6029 | UGGGGUUGUGAUUUCAGGCUU | 21 | 195 |
| bna-miR6030 | UCCACCCAUACCAUACAGACCC | 22 | 99 |
| bna-miR6031 | AAGAGGUUCGGAGCGGUUUGAAGC | 24 | 27 |
| bna-miR6032-5p | UGGAGCAUCAACAGAUCUCGG | 21 | 1 |
| bna-miR6033 | UGAACCAGAUAGAGUGGGACU | 21 | 11 |
| bna-miR6034 | UCUGAUGUAUAUAGCUUUGGG | 21 | 40 |
| bna-miR6035 | UGGAGUAGAAAAUGCAGUCGU | 21 | 14 |
| bna-miR6036 | AUAGUACUAGUACUUGCAUGAUCA | 24 | 3 |
| bna-miR824-5p | UAGACCAUUUGUGAGAAGGGA | 21 | 477 |
| bna-miR860 | UCAAUACAUUGGACUACAUAU | 21 | 77 |

miR_name, miRNA name; Len, length of mature miRNA; Read, the total read count of all the small RNA libraries.

**Table S2 Precursors of 35 novel miRNAs in *Brassica napus***

| miR-name | Precursor sequence |
| --- | --- |
| bna-novel_1 | aucaauugguuuuagguuaagaagcccauaauggagcguauuagacacaaaguggcauaagcaauauaauauagaugggcuuccuccuaacaccaauugauu |
| bna-novel_2 | acucauuaccaucagagccacauaccauguuccguuuaaaacgaauaaaauacguggcauugguaguaaugagugu |
| bna-novel_3 | ucauugagugcagcguugaugugauuuacuuuuuuuauuguugaauggauuaaagcaauuuacaucaauguuggcucaauuaugu |
| bna-novel_4 | uaaggucacugugguaauccaccacagaaguuuaauguuguguuuggcacgggaaguuccuuuuauauauuagaacuucacgugucgaacacaacguuaaacuuuugugguggauuaucgacacugaucucauc |
| bna-novel_5 | ucauugagugcagcguugaugugauuuacuuuuuucauuguugaauggauuaaagcaauuuacaucaauguuggcucaauuaug |
| bna-novel_6 | aaggacucuaaucagaaauauugguccaauggucuuaucuggaauccuuaa |
| bna-novel_7 | ugccuggcucccuguauaccacaagcaaacaccgaucucauuaugaaaucgaucgcuagugguguauagaguagucaagcaug |
| bna-novel_8 | aucucuaauguauaacuccauuuuuuccuccaaaauggaguaaaaguaaaaauggaauaaaauugcuucaauccuacuucauuuuucacuccauaauagagugaugaacaaacaaaaaauagauuacuacauuuauagaguaaauuucauuauagagugagauaugaaguugaguuggagcauuccuuacuucauauucacuuuuacuccauuuuagaggaaaaaauggaguagauauggagaugccc |
| bna-novel_9 | agaguuuccuuaaguccauucacauguugugauuugauccaauuagcuccagacucauucacucacuuaccgagucacuaaaauaggaaugaacucguuacaugaguaaaugaugcgggagacuuauuggaucuuagauucauugcgauuggacugaagggaacucccu |
| bna-novel_10 | uaagaucuuuguacuuucggguuuaacuaauuauggauauuuucggauucuaauuccgauuugauuuguacccaaccccgaaaguacaaagaucugaaa |
| bna-novel_11 | aacaguuggauuggcucuacguggagaaauuuugggaaucuuuaggauuuuguugcauguauucuugguaccuuuuuuuuuuuaacucaaugaguucauaacuuuuucugcaaaagccgaguaacaauauagcauuaaaucguguauuagucaguuuagaaaccaucuuugcuuaagacuuauauacgaaauaaaagcugauuaaagaagaauauaugagagaucagagaaaauuuuggacauaucugcucuacauuauacuuggcgaagguuaaaacgauggaggacaaaacugaugca |
| bna-novel_12 | uuuucagcaaucucuuuuccauuuaccuucguucuaaagguuccacucccaaaaauuguaaugcccugaugagauggcgucaaggaaaaugggaaagauuguugaucaga |
| bna-novel_13 | uaaaguagagcucggugacggagcuauauaugaggguacaauaggaagggaaacauuaacguauaggacaggcuauaggggcuuuagcaccauagaugacguuuauuuugggguugggacgaaacuaagaaugaucccuacuugaacccgcuucuuaguuucguccccaaccccaaaauaaacgucaucuauggugcuaaagccccuauagccuguccuauacguuaaugguucccuuccuauuguacccucauauauaacuccgucaccgagcucuacuuuaua |
| bna-novel_14 | ucgcuucuguugaauaauuuugacaaaaaauuauagggcauacauuuauauauuuaauuuuauauauacauagacaaacuuuguugauuccuuaacuagauuacauagaucaacaaaucacaaucaauguuauuuucacauauuuucuaguuaaggaaucaacaaaguuugucuauguauauauaaaauuaaauauauaaaugucugcccuauaauuuuuugucaaaauuauucaacagaagcgaau |
| bna-novel_15 | auaugaggguacaauaggaagagagacauuaacguauaggacaggcuaggggcuuuagcaccauagaugacguuuauuuugacguuuacuugaaccccuucuuaguuucgucuccaaccccagaauaaacgucaucuauggugcuaaagccccuauagccuguccuauacguuaacguuucccuuccuauuguacccucauauauagc |
| bna-novel_16 | auguuuauuguaggucuuuuagguugaauuuuuuagcgauacggucuuuuagcuuuuaacuaaaaaagcuaagagacgucucuuauaucucuuauuuaagagacgucucuuauaucucuuauuuaagagacgucucuuagcuuuucuaguuaaaagcuaagagaccguaucuuauauuacgauaagaaauccaaccuaagagaucuguaauaaacaugc |
| bna-novel_17 | acgaacacugaguaauaucuggauaacuauuuucuuaauaacaccacuaucagcauacauuauugaaacauauaguaaguugaaguaauauauauauauauauauauauauauauauauauauauauauauauauauuguacaacuuauccauuuuuggcacacuucaacuuacuauauguuucaauaauguaugcugauagugguguuauuaagaaaauaguuauccagauauuacucaguguucguug |
| bna-novel_18 | guuguacauuguacacagcgguguacgccuccguguaugccaaguguacgcuccaguguacgcccuaguuuacgcuccgguacgccccgguguacgcccugguguacgcccccguguacgcccccguguacucacugguuuacgcuccgguguacgccccccguguacgcuuucauguaugcuacaguguaugcuacgguguauuucuuaguguacgcuaugguguacacugcagugcacuguacauugc |
| bna-novel_19 | uugcaaacugaauuaugagucuaugacuuuuaaaaaucuuauauauauaguuguuuauaauagucauagacucauaauucaguuugcaauc |
| bna-novel_20 | aagauacggucucuuaacuuuuagcuaaaaaaaacuaagagacgucucuuaaauaagauauauaauauaagagacgucucuuagcuuuuuuuaguuaaaaguuaauagaccguaucuuaua |
| bna-novel_21 | aacgaucuuguuugguuuugaagaaaaaccuagggaacauuuacuuuuaaagcugaacaguugauuuaguaaaaacccuacaauuuaguaaaaacuguucaguuuuaaaagcaacuguuuccuagguuuaucuucaaaaccauacaagaucguuuu |
| bna-novel_22 | ugaagcugccagcaugaucuaguuaacuuuauuucuccguuguuuauccaugacaauggaaaagggauaagugucgauuagaucauguucguaguuucacc |
| bna-novel_23 | accucuaaaacacacaagaagaagcagcagaacucacaugaacggacagaccggcugcugcuuuuucuucuuguguauucugcugcuucuucuugugcguuuauagguag |
| bna-novel_24 | uguuucgcuguuacucaugcaucaucuuucuccauuguucuucauuucucucucuucucugcuacuaaaaacagagacgcgcgucauccauucuucucucuuucuuuuuggauacucuuuuucccuucuaaauuuugaucuguaguuacugcggcagcguucguuuaacgaacaggacuaauaauguugucguguuuuugaaaacagaggagaaagaugaugcaugaguaacagcgaaacaaa |
| bna-novel_25 | ccauaaaaaggguucacaaaguauuuuuuuauuauuuuuuuuucuguuugauuuuuguuuuaaaaaaaaaaaauuauuaaucggaccaaucgcgggccgccacgugucguggggcccgcgcuacagugaugauccguguucagugcagugaacucccaagaggcagguucauugcuuuuauuuauuuuaauuuuuuuuuuuuucgaaaacugugugaacucuccauggag |
| bna-novel_26 | aagugcuaccgguauccacgugaagauuacaggaggugaaccaaacuagauuuugguuauuaacgcaagcccuucccgcaaaggacuuguguuuauagcggaaaucuaguuuggugugguucaccuuguguaaaccaauaucuucuucacguugauacauguagcucuuug |
| bna-novel_27 | uuaaucguuuugugacucuuuuacacauaaagcauaaaauuuguggguuucuauguuuacguguaaaauaguuacaaaacgauuagugc |
| bna-novel_28 | ucauugagugcagcguugaugugauuuacuuaucuuuuucauuguugaauggauuaaagcaauuuacaucaacguuggcucaauuaug |
| bna-novel_29 | uucagcuggguacgagccaccaggccauguggaucugagaggcuuugauccguuauccguccacugccgagagaaggaggugaucgcugcucugugaaaccagccucacgcauugcccuccccucuuuaggaaccgccugagucaagccuucgccuaacacaaacggaucacucucggcuuggacugggcgguccagaagaugccagacagaaccgaccggugggccgagccaguguuccaccaucuuauuguccccacaugggucacaaacucuuguuacugagcucgacg |
| bna-novel_30 | aucugcaucgagugaacucuaugggaaguucacauaguuuaaaaaaaaauuaaaauaaacaaaaaaagugaaccuguuuuuuaggaguucacugcacugaacucagaucaucacuguagcgcgggccccacggcacauggcgguccgcgauugguccaauuaauuaauuuuuuuuuuaaaacaaaaaucaaacagaaaaaaaauauaauaaaaaaauacuuuuucauggaauucacugaugcagaugcu |
| bna-novel_31 | uucuugugguuguagagucuugucaucgagaauuugcuggcucuguuucuagauauucaaacuauuggugauagaaacauguuaaagcuugcaaauccucuaugacaagacucuacaacaucagaaac |
| bna-novel_32 | cggauuuuagcugcguagcuaaugaaaaaucauucaaaggaaugaagggugauuguucauuggcuacgcugcugaauccgc |
| bna-novel_33 | ccuucccaaaauucuacaauuagaauuaaacuauaucuuguaauuguagaauuuugggaagggc |
| bna-novel_34 | acuuugaaacuuugaucuagaucuaacauuugguaucaaagcuuucacuccauaauaaagcucugauaccaaauuguuagcacuagaucaaagcuuuaaugu |
| bna-novel_35 | cauuuacagaucgaagacauuugguuuugaagaacaaaugaagauaauuaaaacaaaauguuuucgaucuguaaauuu |

miR_name, miRNA name

**Table S3 Target genes predicted by psRNATarget in *Brassica napus***

| miRNA | Predicted targets using psRNATarget |
| --- | --- |
| bna-miR390a/b/c | BnaA07g14240D,BnaC05g05330D,BnaCnng40710D,BnaC07g38750D,BnaC05g00720D,BnaA10g00660D,BnaA06g09370D,BnaC05g49670D,BnaA07g02210D,BnaC02g07070D,BnaA02g03430D,BnaCnng77670D,BnaA05g07920D,BnaA10g27490D,BnaA09g21950D,BnaA01g02120D,BnaA01g15760D,BnaC01g18810D,BnaC09g24170D,BnaC05g10700D,BnaA06g17430D,BnaA07g04740D,BnaCnng00430D,BnaCnng42950D |
| bna-novel_4-3p | BnaC03g50530D,BnaA01g13710D,BnaA06g22940D,BnaA08g10700D,BnaC03g65310D,BnaA07g03150D,BnaC01g01700D,BnaC01g16110D,BnaC06g32890D,BnaC09g33510D,BnaA10g29360D,BnaAnng05680D,BnaC03g20860D,BnaA03g17340D,BnaC09g25570D,BnaA06g10190D,BnaA08g22150D,BnaC03g45740D,BnaC03g34440D,BnaC09g19900D,BnaC04g15240D,BnaA04g04910D,BnaCnng73250D,BnaA10g03990D,BnaA09g34280D,BnaC01g39030D,BnaA01g32120D,BnaC05g04110D,BnaAnng11740D,BnaA08g13200D,BnaC05g20620D,BnaC04g26900D,BnaC04g32030D,BnaC03g58760D,BnaC04g09730D,BnaA07g24090D,BnaC07g45830D,BnaA09g49680D,BnaA07g31000D |
| bna-novel_31-5p | BnaA02g13640D,BnaA09g53400D,BnaA09g17760D,BnaC09g18620D,BnaCnng13540D,BnaC04g02290D,BnaC03g13170D,BnaA05g12410D,BnaA01g15140D,BnaC07g49310D,BnaA02g23420D,BnaC03g59630D,BnaC01g22250D,BnaC02g46850D,BnaCnng55230D,BnaC05g18790D,BnaA09g26820D,BnaC02g10670D,BnaC03g14400D,BnaA03g11620D,BnaC03g71620D,BnaA01g23710D,BnaC02g45430D,BnaA02g12920D,BnaC05g40390D,BnaA05g26260D,BnaA09g19450D,BnaC09g27740D,BnaC09g51780D,BnaC02g17990D,BnaA02g08000D,BnaC03g30250D,BnaA03g25740D,BnaC03g77730D,BnaA08g02030D,BnaA02g25240D,BnaA02g25230D,BnaA04g27110D,BnaC04g50950D,BnaC04g00770D,BnaA05g01210D,BnaA07g30580D,BnaC08g31510D,BnaA06g18060D,BnaC03g55910D,BnaC01g02910D,BnaA01g01780D,BnaC08g36310D,BnaA09g43680D,BnaA01g26960D,BnaA06g18430D,BnaC09g49900D,BnaA05g28040D |
| bna-novel_34-5p | BnaCnng11600D,BnaUnng05200D,BnaC04g54900D,BnaA05g33920D,BnaC02g28900D,BnaC06g37650D,BnaC08g20400D,BnaCnng68810D,BnaCnng47220D,BnaA06g26570D,BnaC07g30390D,BnaC09g18980D,BnaA01g17700D,BnaC03g66530D,BnaC09g47950D,BnaC05g10730D,BnaC06g43220D,BnaA04g02780D,BnaCnng20400D,BnaC04g24820D,BnaA02g25200D,BnaAnng15440D,BnaC08g42220D,BnaC05g30720D |
| bna-novel_1-3p | BnaC03g58260D,BnaC03g12860D,BnaC03g43810D,BnaA03g37390D,BnaA08g02880D,BnaAnng05800D,BnaC04g40330D,BnaA04g17020D,BnaA03g10180D,BnaA03g38370D,BnaC09g28490D,BnaC07g33590D,BnaA09g22160D,BnaC09g46290D,BnaA01g20710D,BnaCnng22500D,BnaA06g16100D,BnaC08g20870D,BnaAnng29380D,BnaA08g02730D,BnaC07g18680D,BnaA05g24600D,BnaC09g35520D,BnaA10g13040D,BnaA08g18610D,BnaA04g11080D |
| bna-miR158-3p | BnaC04g20450D,BnaA02g26990D,BnaC02g33900D,BnaA09g41930D,BnaC08g34430D,BnaC04g35970D,BnaA04g13700D,BnaA07g13010D,BnaC04g45800D,BnaC08g14430D,BnaA10g18020D,BnaC05g05730D,BnaA07g20970D,BnaC06g20970D,BnaC09g32230D,BnaA03g33120D,BnaA03g33090D,BnaC09g12580D,BnaCnng21450D,BnaC09g11440D,BnaA09g11120D,BnaA02g26710D,BnaA09g46030D,BnaA10g07840D,BnaA06g32750D,BnaA05g01260D,BnaC04g00830D,BnaC06g03890D,BnaA06g02640D,BnaC07g23740D,BnaAnng15460D,BnaC02g19490D,BnaA02g14500D,BnaA03g41620D,BnaC05g05460D,BnaA03g05920D,BnaC09g41510D,BnaA06g04520D,BnaA05g03640D,BnaA06g35310D,BnaA03g21490D,BnaC03g25870D,BnaC04g33980D,BnaC05g19760D,BnaA09g29450D,BnaC06g42540D,BnaC05g44930D |
| bna-novel_28-3p | BnaA03g44280D,BnaC02g01480D,BnaA08g23780D,BnaC07g36070D,BnaC01g36570D,BnaC09g03610D,BnaA03g35900D,BnaA09g04200D,BnaC09g03630D,BnaA09g04230D,BnaA09g29630D,BnaA06g25030D,BnaC05g19420D,BnaA10g03000D,BnaC01g23720D,BnaC08g38730D,BnaC05g48730D,BnaA05g33960D,BnaA02g31100D,BnaA08g27430D,BnaC08g00240D,BnaA03g52590D,BnaA05g36380D,BnaC05g25820D,BnaA02g03360D,BnaA09g56610D,BnaAnng29260D,BnaA03g17930D,BnaC03g21490D,BnaA03g18730D,BnaA09g15720D,BnaA01g05240D |
| bna-miR408-5p | BnaC03g47260D,BnaCnng05600D,BnaA03g14130D,BnaC03g17100D,BnaA03g06080D,BnaC03g06470D,BnaC08g32460D,BnaA09g40120D,BnaAnng32310D,BnaA03g44580D,BnaC03g43270D,BnaCnng02530D,BnaA01g21380D,BnaC01g26800D,BnaA07g18490D,BnaC05g32850D,BnaA10g09090D,BnaC09g31260D,BnaC03g30170D,BnaA03g37030D,BnaC09g19110D,BnaAnng29100D,BnaC05g27080D,BnaC05g38100D,BnaC02g46330D,BnaC08g02540D,BnaC02g28510D,BnaCnng23430D,BnaA02g21480D,BnaA03g22490D,BnaA04g13930D,BnaC02g22520D,BnaA02g16600D,BnaC05g08830D,BnaA10g27200D,BnaCnng03530D |
| bna-miR398a-3p | BnaC02g09710D,BnaC09g33240D,BnaA10g10840D,BnaA02g08520D,BnaA03g10640D,BnaC03g13330D,BnaC02g12070D,BnaC08g48700D,BnaC07g17640D,BnaA05g28040D,BnaC08g12150D,BnaC01g18280D,BnaA01g15390D,BnaA09g30380D,BnaC08g01260D,BnaA08g28240D |
| bna-miR394a/b | BnaC07g11360D,BnaA07g08860D,BnaC05g21250D,BnaA09g27990D,BnaC06g37980D,BnaA07g33430D,BnaC05g00640D,BnaA10g00570D,BnaC06g12020D,BnaAnng27520D,BnaA09g12550D,BnaC05g42180D,BnaA06g18900D,BnaC09g36350D,BnaA10g13800D,BnaC07g26320D,BnaC01g34630D,BnaA09g55110D,BnaC08g29300D,BnaC08g29300D,BnaC06g16620D,BnaA07g17890D,BnaA09g37460D,BnaA07g36430D,BnaA03g26160D,BnaC06g29360D,BnaA06g27200D,BnaA10g28990D,BnaC05g10580D,BnaA08g27890D,BnaCnng22220D,BnaC08g00690D,BnaA09g43170D |
| bna-novel_33-3p | BnaC04g24560D,BnaC09g02160D,BnaA01g00880D,BnaC03g12250D,BnaA07g38350D,BnaCnng16460D,BnaA06g05260D,BnaC05g06550D,BnaCnng41780D,BnaC09g35020D,BnaC01g15190D,BnaA01g13180D,BnaC09g48940D,BnaC06g34770D,BnaA07g31100D,BnaA09g02710D,BnaA03g26920D,BnaC03g31880D,BnaC04g15620D,BnaAnng39120D,BnaC03g73700D,BnaA03g32570D,BnaA06g08340D,BnaC09g49830D,BnaC06g40650D,BnaA08g08370D,BnaC08g48130D,BnaCnng56940D,BnaA01g08480D,BnaC05g27340D,BnaC01g32020D,BnaC02g29770D,BnaC06g37940D,BnaC01g24140D,BnaA01g20810D,BnaC09g08120D,BnaA04g22850D,BnaA02g30090D,BnaA02g30080D,BnaA03g14540D,BnaA02g24540D,BnaC02g32310D,BnaA04g03020D,BnaC01g12860D,BnaA02g35240D,BnaA04g27110D,BnaC04g50950D,BnaC04g00770D,BnaA05g01210D,BnaC09g44050D,BnaA10g20240D,BnaA07g12740D,BnaA07g31360D,BnaC09g51490D,BnaC09g18120D,BnaCnng10780D,BnaA01g36400D,BnaCnng19710D,BnaA05g06000D,BnaC08g03000D,BnaC01g01880D |
| bna-miR159a | BnaCnng51960D,BnaAnng27960D,BnaA02g09340D,BnaC08g30650D,BnaA09g38510D,BnaA07g18670D,BnaC06g42760D,BnaA04g18810D,BnaC04g43020D,BnaC09g31560D,BnaAnng23930D,BnaC01g19500D,BnaA01g16350D,BnaC03g75050D,BnaA06g20460D,BnaA07g12970D,BnaC03g26620D,BnaC04g17160D,BnaCnng31260D,BnaC03g18870D,BnaA03g15690D,BnaC05g44630D,BnaC07g18240D,BnaAnng05340D,BnaA01g06030D,BnaA03g11630D,BnaC03g71630D,BnaCnng06560D,BnaA01g06260D,BnaC04g03750D,BnaC01g07290D,BnaC07g10610D,BnaA06g28740D,BnaA03g04070D,BnaA05g30270D,BnaA10g02010D,BnaA06g33690D,BnaC01g28220D,BnaC01g41960D,BnaAnng11030D,BnaC07g04630D,BnaCnng64820D,BnaA07g03930D,BnaC05g43690D,BnaC02g41080D,BnaC04g35390D,BnaC01g01270D,BnaA03g36730D,BnaAnng36450D,BnaC06g41970D,BnaA03g36750D,BnaAnng39060D,BnaC04g03740D,BnaC03g42930D,BnaA02g07080D,BnaA07g08570D,BnaA01g03320D |
| bna-novel_9-3p | BnaC04g03750D,BnaC04g13290D,BnaA05g11650D,BnaA05g11640D,BnaC01g36430D,BnaC01g07240D,BnaA01g05980D,BnaA05g24520D,BnaCnng59700D,BnaA01g29050D,BnaA03g33680D,BnaC03g38850D,BnaC04g00500D,BnaA05g00870D,BnaC03g36360D,BnaA08g01260D,BnaCnng51960D,BnaAnng27960D,BnaA07g12970D,BnaA03g22590D,BnaC03g26620D,BnaC04g17160D,BnaCnng31260D,BnaC04g03730D,BnaA01g09160D,BnaA08g17500D,BnaCnng34520D,BnaC01g40850D,BnaC07g35270D,BnaC05g23130D,BnaA03g58260D,BnaA09g26360D,BnaA10g19930D,BnaA02g01580D,BnaC04g51140D,BnaC06g26230D,BnaC09g30830D,BnaC04g39930D,BnaA04g16610D,BnaAnng11470D,BnaC02g39490D,BnaC09g50740D,BnaC05g46420D,BnaA05g31830D,BnaA04g01370D,BnaC04g22400D,BnaC06g17680D,BnaC04g49630D,BnaA04g25720D,BnaC09g20690D,BnaC07g30340D,BnaAnng19210D,BnaA09g32590D,BnaC08g38010D,BnaA09g13960D,BnaCnng11880D |
| bna-novel_3-3p | BnaA10g03000D,BnaC04g49180D,BnaA04g25320D,BnaA06g02280D,BnaA08g05320D,BnaA10g13960D,BnaC09g27690D,BnaC05g37990D,BnaC09g27620D,BnaCnng54760D,BnaA10g28720D,BnaA06g25030D,BnaA09g02040D,BnaA09g02030D,BnaCnng38170D,BnaC04g46930D,BnaA04g22430D,BnaA08g23780D,BnaA09g47920D,BnaC08g42210D,BnaA03g25700D,BnaC03g30220D,BnaA03g44280D,BnaCnng01410D,BnaA01g22800D,BnaC01g36570D,BnaC08g09580D,BnaA02g23580D |
| bna-novel_5-3p | BnaA10g03000D,BnaC04g49180D,BnaA04g25320D,BnaA06g02280D,BnaA08g05320D,BnaC09g27690D,BnaA10g13960D,BnaC05g37990D,BnaC09g27620D,BnaA06g25030D,BnaCnng54760D,BnaA08g23780D,BnaA10g28720D,BnaA09g02040D,BnaA09g02030D,BnaCnng38170D,BnaC04g46930D,BnaA04g22430D,BnaA09g47920D,BnaC08g42210D,BnaA03g25700D,BnaC03g30220D,BnaA03g44280D,BnaC08g09580D,BnaA02g23580D,BnaCnng01410D,BnaA01g22800D,BnaC01g36570D |

**Table S4 All the primers used for qRT-PCR and 5^,^modified RACE analysis**

| **Primer names** | **primer sequence(5^,^-3^,^)** | |
| --- | --- | --- |
| miR158-stem-RT | | GTCGTATCCAGTGCAGGGTCCGAGGTATTCGCACTGGATACGACTGCTTT |
| miR158-Forward | | CGGCGGTTTCCAAATGTAGAC |
| miR159-stem-RT | | GTCGTATCCAGTGCAGGGTCCGAGGTATTCGCACTGGATACGACTAGAGC |
| miR159-Forward | | CGGCGGTTTGGATTGAAGGGA |
| miR398-stem-RT | | GTCGTATCCAGTGCAGGGTCCGAGGTATTCGCACTGGATACGACCAGGGG |
| miR398-Forward | | CGGCGTGTGTTCTCAGGTCA |
| miR827-stem-RT | | GTCGTATCCAGTGCAGGGTCCGAGGTATTCGCACTGGATACGACTATTTG |
| miR827-Forward | | CGGCGGCTTAGATGACCATCAA |
| Novel34-stem-RT | | GTCGTATCCAGTGCAGGGTCCGAGGTATTCGCACTGGATACGACTCTAGA |
| Novel34-Forward | | CGGCGGCACTTTGAAACTTTGA |
| miRNA-universal-reverse-primer | | GTGCAGGGTCCGAGGT |
| U6-Forward primer | | TTGGAACGATACAGAGAAGATTAGCA |
| U6-Reverse primer | | TTGGACCATTTCTCGATTTGTG |
| myb33-Forward-primer | | TCGTCATCTCCTCCACACTCTG |
| myb33-Reverse-primer | | CCTCGGATTTAGTTTGGGATAC |
| myb65-Forward-primer | | CTTCCCCAAAGCAAATCTG |
| myb65-Reverse-primer | | TTCACTGCCCCAAACAAG |
| ACTIN2-qRT-Forward primer | | CCAGAAGGATGCATATGTTGGTGA |
| ACTIN2-qRT-Reverse primer | | GAGGAGCCTCGGTAAGAAGA |
| CSD1outer primer | | TCAGTGATTGTGAAGGTGGCAGTT |
| CSD1inner primer | | ATTAGCATCCTCAGGTGCACCG |
| PPR outer primer | | CAGAAACCAACTATCATACAGTTGT |
| PPR inner primer | | GGTAAATAAATTAGGAAAGACTCC |
| MYB outer primer | | CTTCACTATCGTTGGCTCATTGTA |
| MYB inner primer | | GATGATTGATGAGAAGAGTTTAGA |

**Table S5 The sequences used for the construction of *MIR159* over-expressed vectors**

| pri-miR159 | Sequence |
| --- | --- |
| pri-miR159-chrA7 | CCCGGGTACTCAGGTCAGATCCACAACATAGGTGGATTTTTTGATTTGACAAACATGTTTATCTGGATAGATCTTTGGTCTTCCAAATAATTTTCTTGAGAACAAAAAAGAAGATTGAAAGTAGAGCTCCTTTAAGTTCAAACGAGAGTTTAGTAGGGTAAAGAAAAGCTGCTAAGCTATGGATCCCATAAGCCCTAATCCTTATAGAGAGAAAAAAAGGATTTGGTTATATGGCTTGCATATCTTAGGAGCTTTAACTTGCCCTTTAATTGCTTTTACTCTTCTTTGGATTGAAGGGAGCTCTACAACTTCTTTCTCTTCATTTAAATTTAATTATCTACAAGAATGATTGAATTGTTTGTATGATTGAATTGCTTGTTTTCCAGGCTTTTCTGTGTTCACAAGCATTCTTCTAGAGGATGTTCCTCTGGTTAATTTTCCGATAGTGTCTTTCTGTGGTGATTGTCGAC |
| pri-miR159-chrC6 | CCCGGGGTTACTCATGTCAGATCCACAACATAGGTGGATTTTTGATTTGCAAGCATCTTTATTTGGATAGATCTTTGGCCTTCAAAATAATTTTCTTGAGAACAAAAAAGAAGATTGAAAGTTGAAAGTAGAGCTCCTTTAAGTTCAAACGAGAGTTTAGTAGGGTAAAGAAAAGCTGCTAAGCTATGGATCCCATAAGCCCTAATCCTTATAGAGAGAAAAAAGGATTTGGTTATATGGCTTGCATATCTTAGGAGCTTTAACTTGCCCTTTAATTGCTTTTACTCTTCTTTGGATTGAAGGGAGCTCTACATCTTCATTCTCTCCATTTAAATTTAATTATCTACATGAATGATTGAATTGTTTGTTTTCCAGGCTTTTCTGTGTTCATAAGCATTCGTCTAGGTCGAC |

Note: The “CCCGGG” and “GTCGAC” are the restriction endonuclease sites of *Sma* I and *Sal* I, respectively.
