# Supplementary material for: Identification of miRNAs and their target genes in genic male sterility lines in Brassica napus by small RNA sequencing
Source: BMC Plant Biol. 2021 Nov 9;21:520. doi: 10.1186/s12870-021-03306-w (PMC8576947; doi:10.1186/s12870-021-03306-w)
Supplement: Supplementary file 2 — Additional file 2: Figure S1. The secondary structures of new conserved miRNAs and novel miRNAs identified in Brassica napus. [file 12870_2021_3306_MOESM2_ESM.pdf]

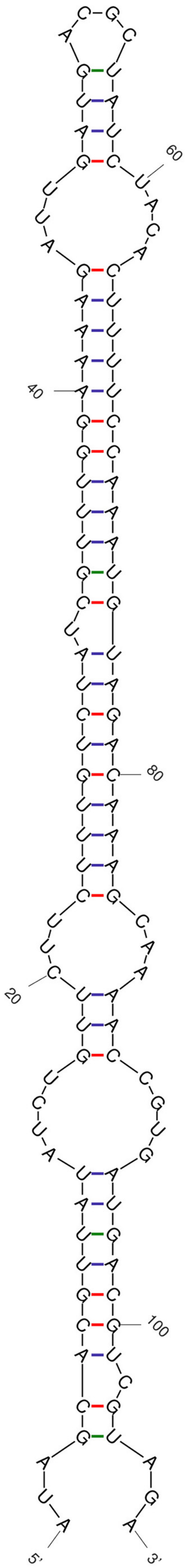

bna-miR158.1

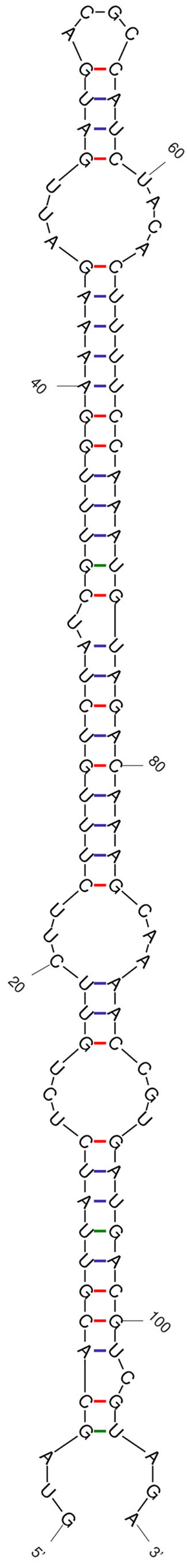

bna-miR158.2

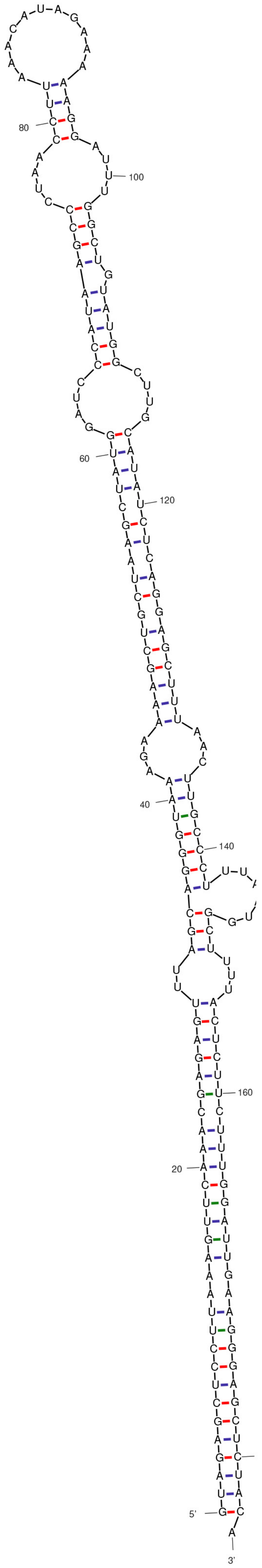

bna-miR159b.1

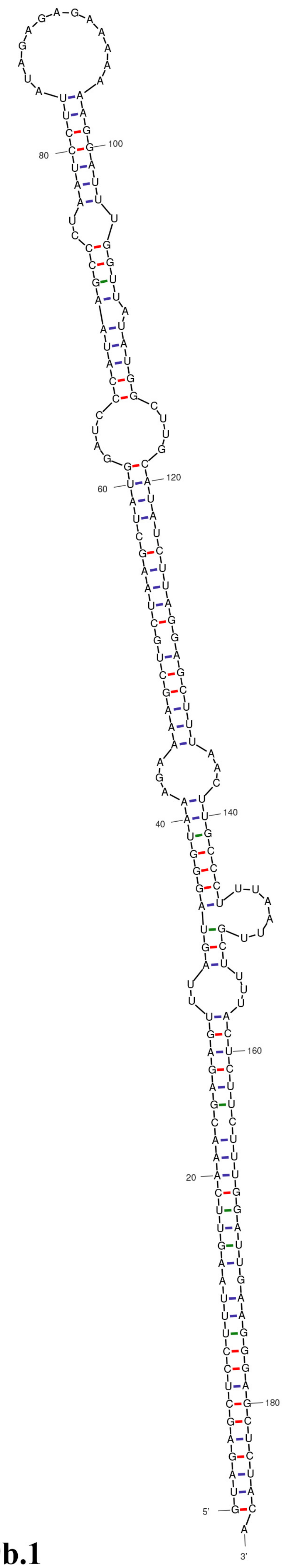

bna-miR159b.2

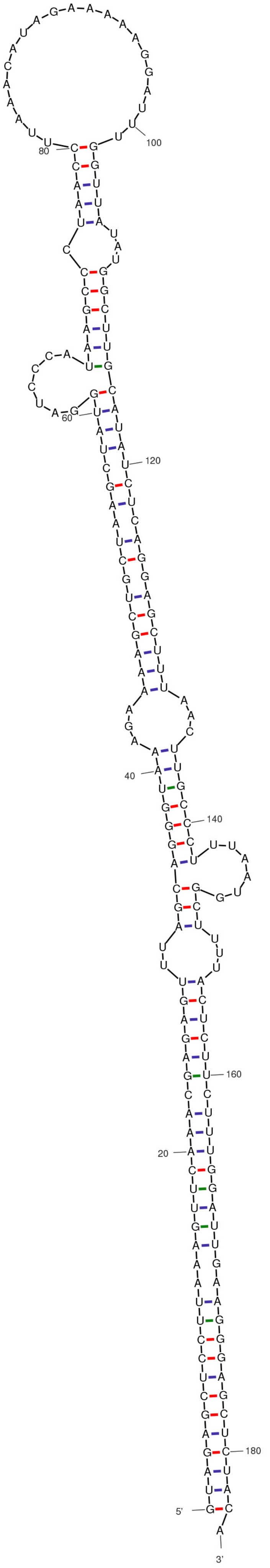

bna-miR159b.3

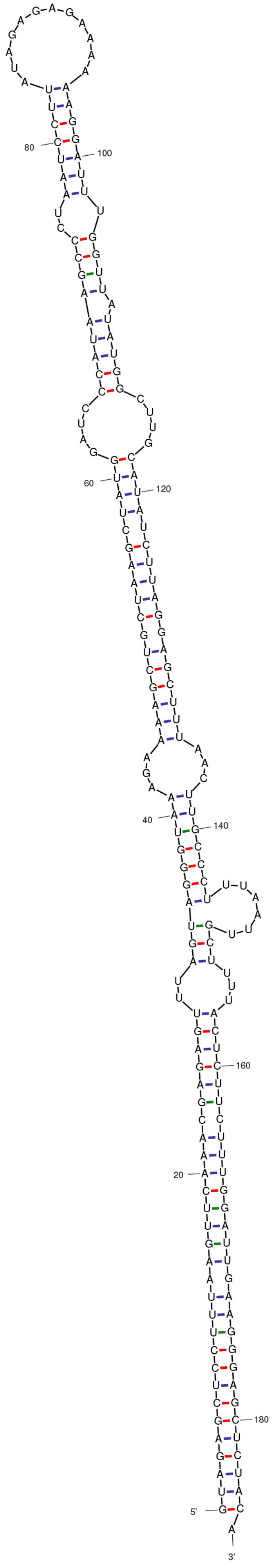

bna-miR159b.4

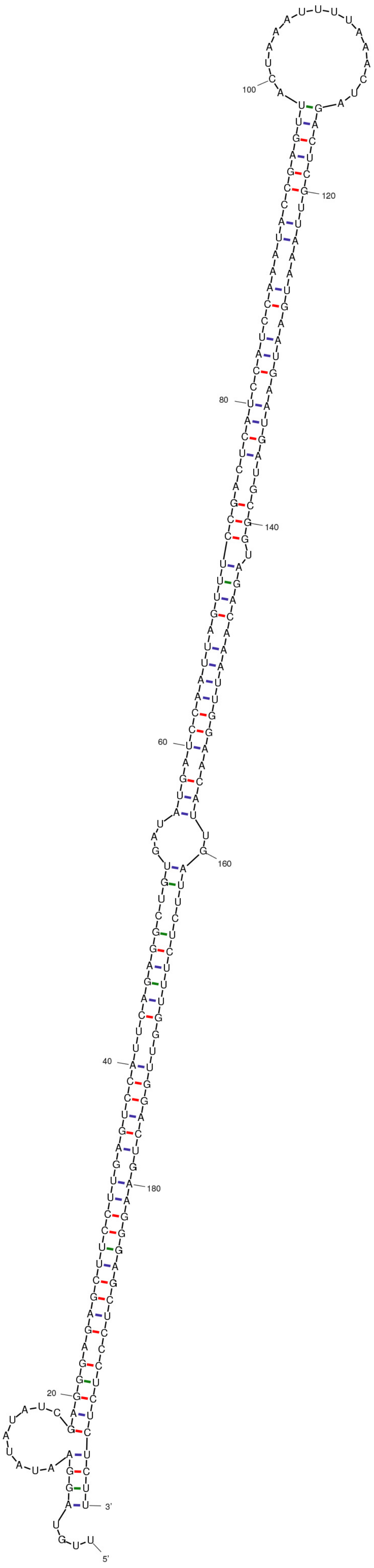

bna-miR319a

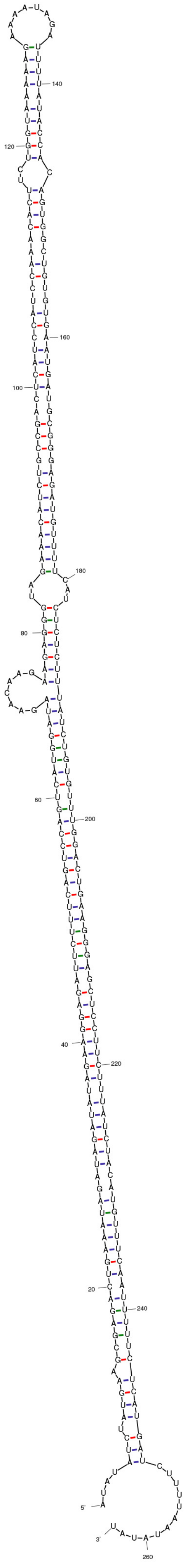

bna-miR319b

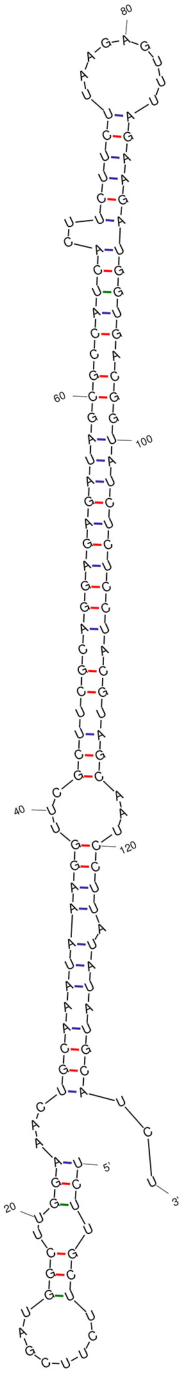

bna-miR391

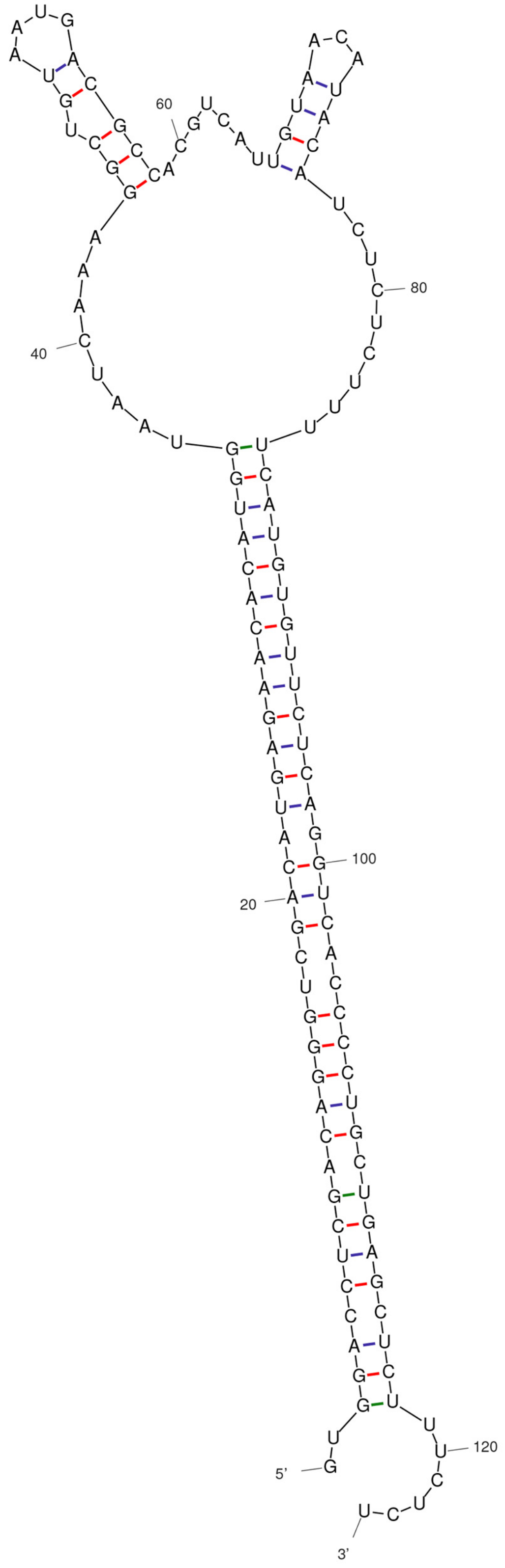

bna-miR398a

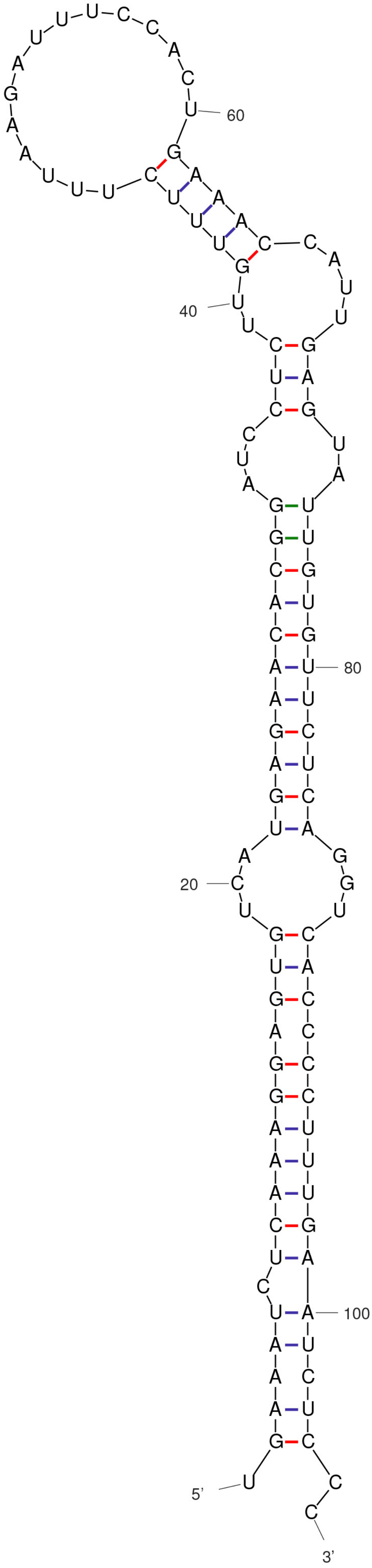

bna-miR398b

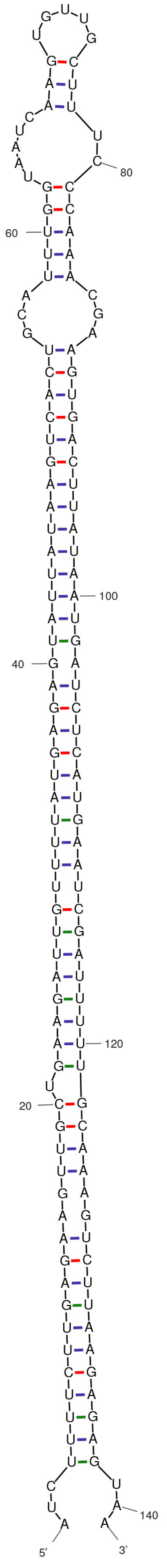

bna-miR400

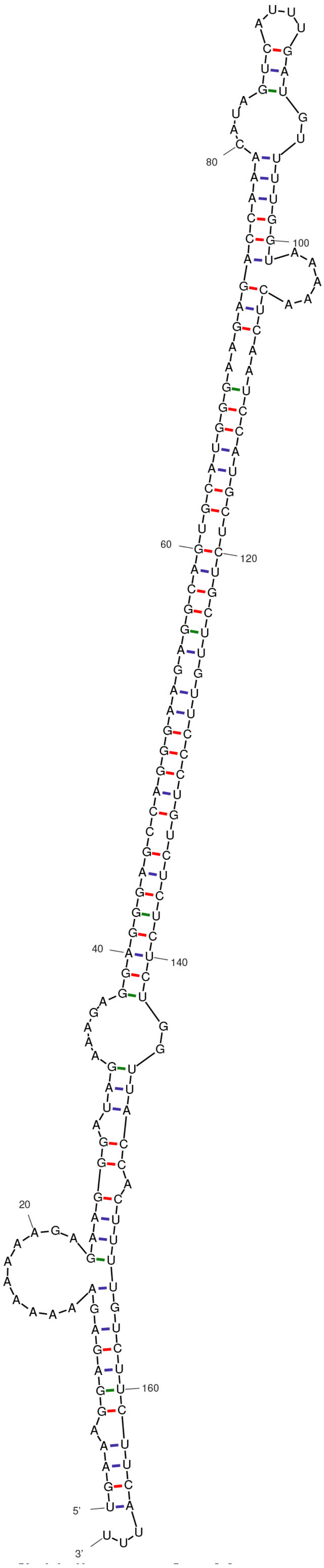

bna-miR408a.1

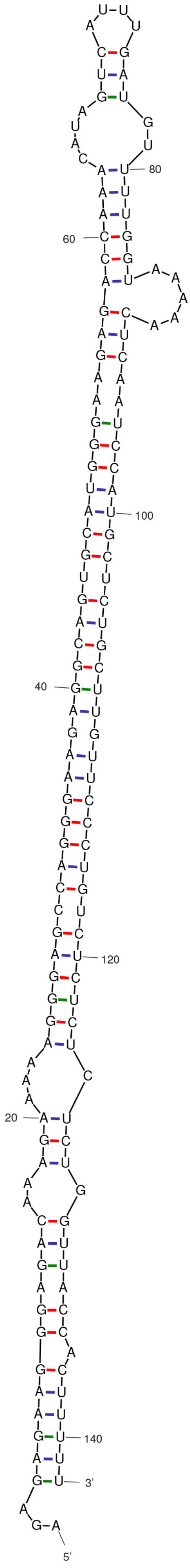

bna-miR408a.2

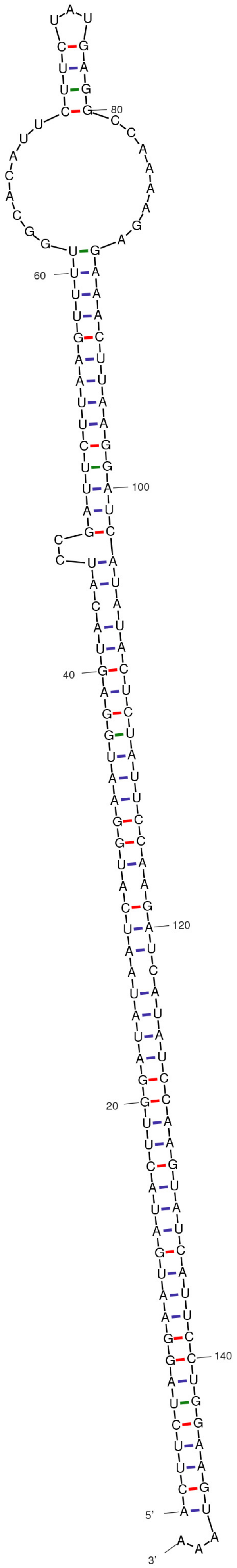

bna-miR9554

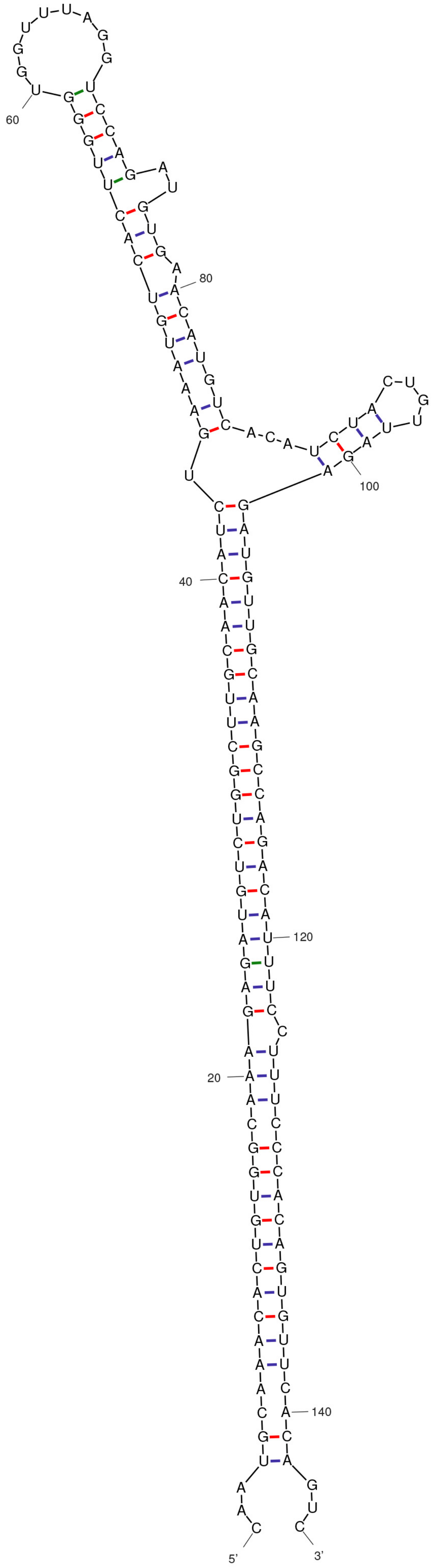

bna-miR9558

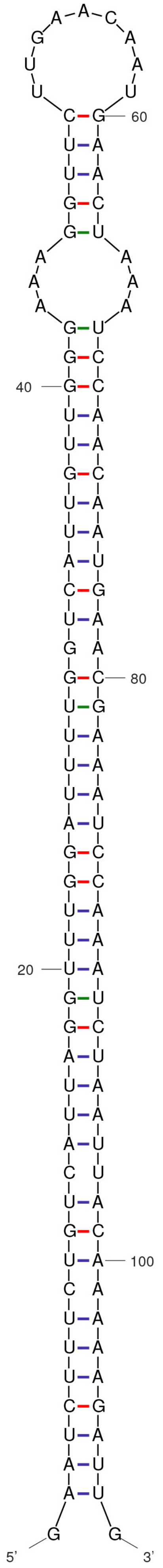

bna-miR9559

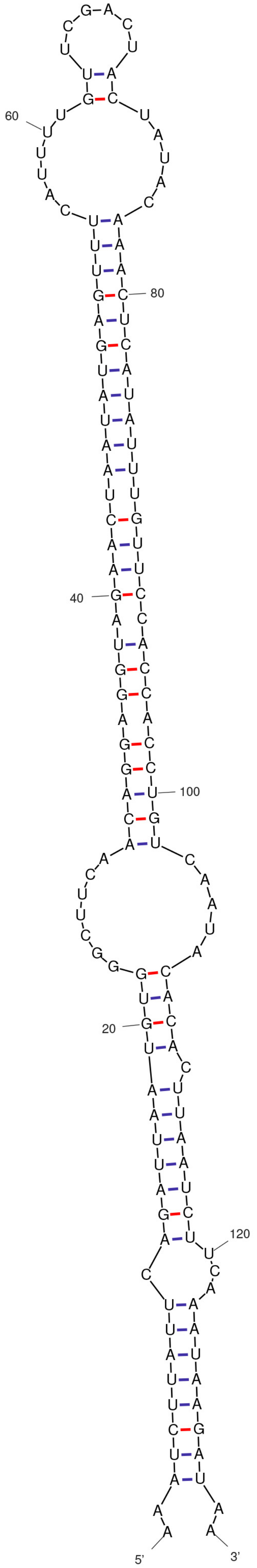

bna-miR9560a

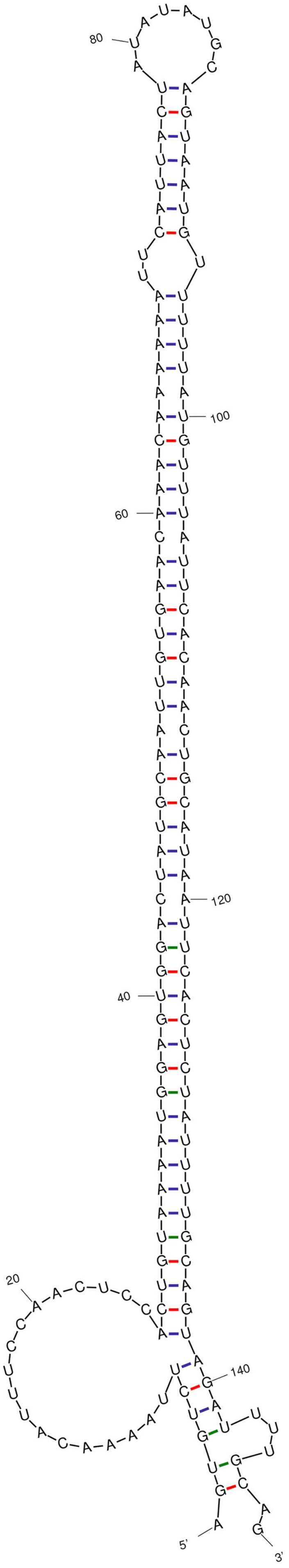

bna-miR9562

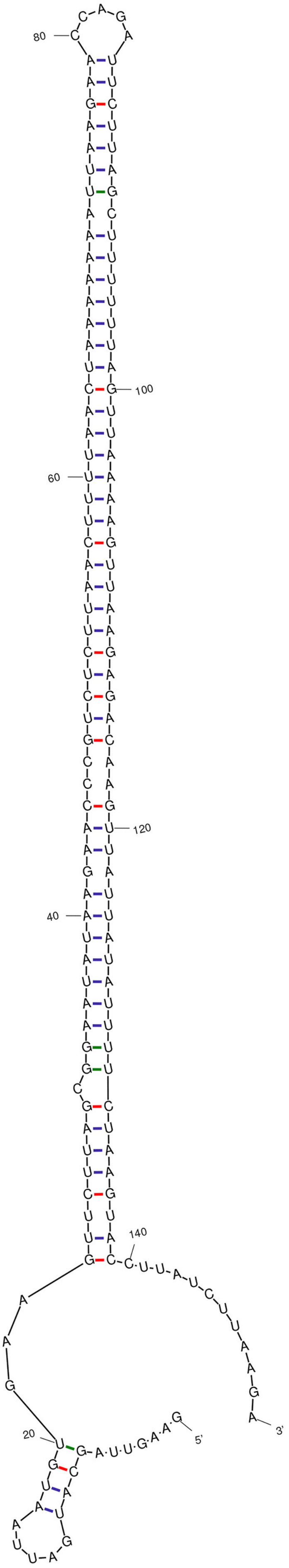

bna-miR9563a

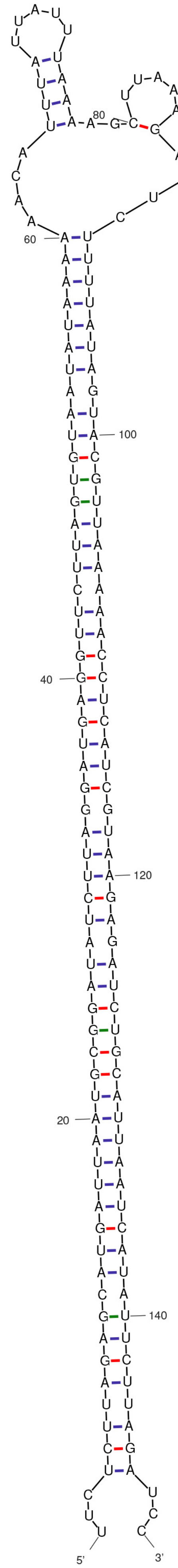

bna-miR9568

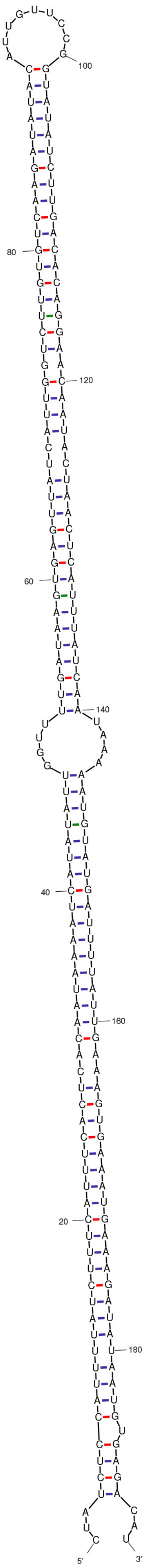

bna-miR9569

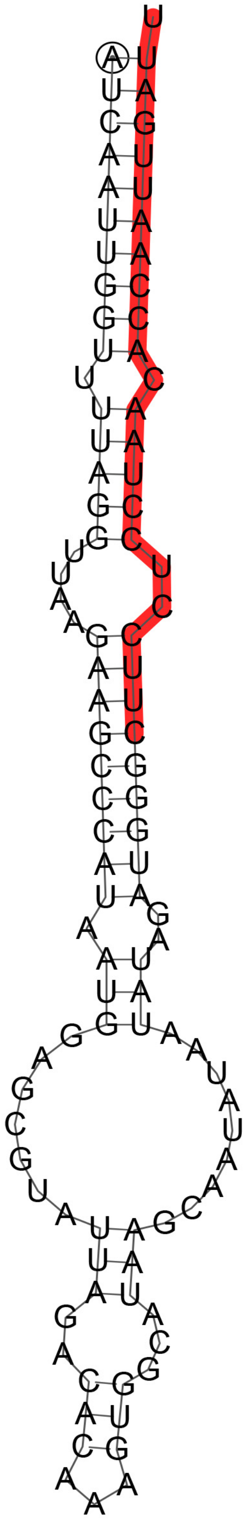

bna-novel_1

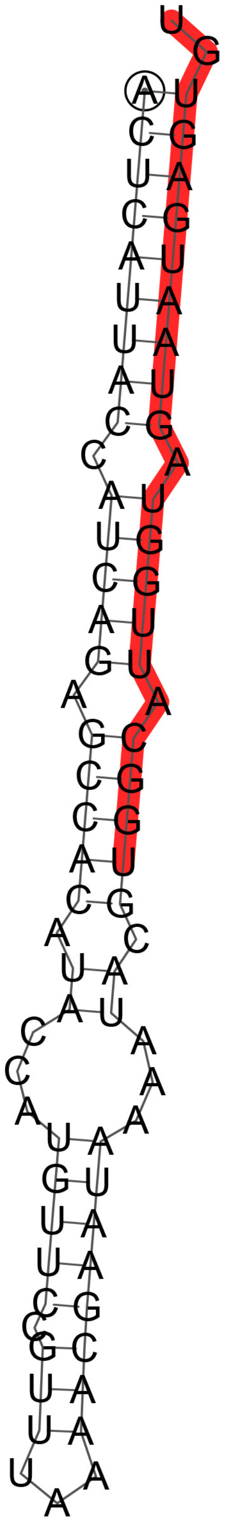

bna-novel_2

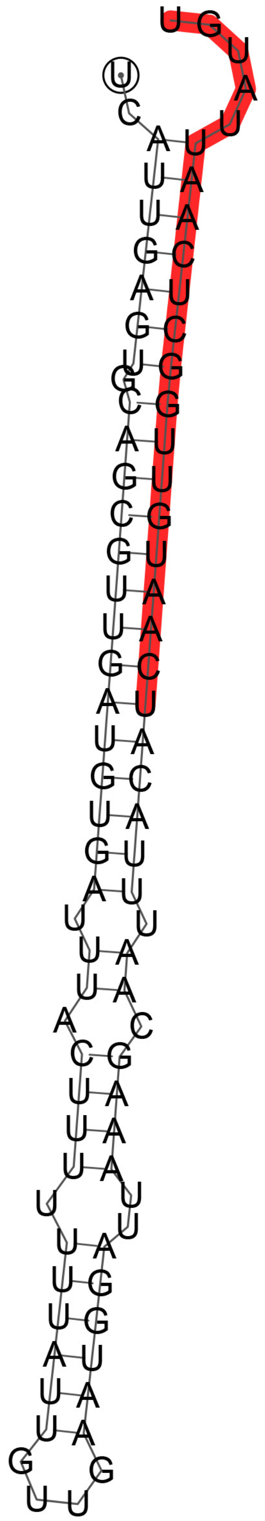

bna-novel_3

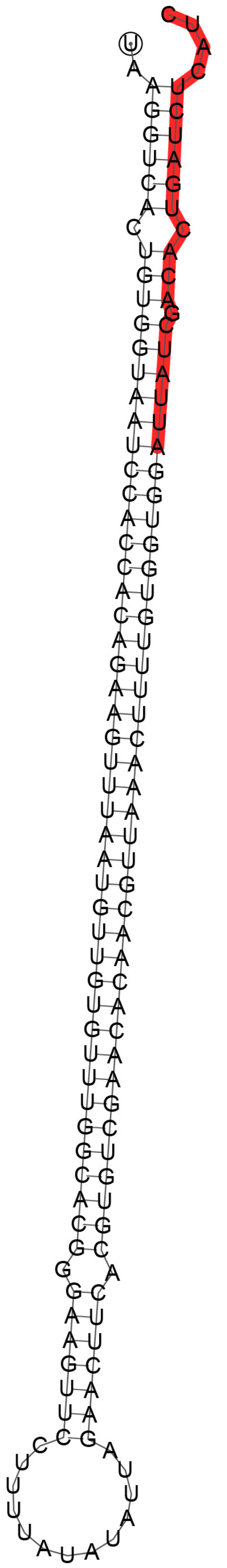

bna-novel_4

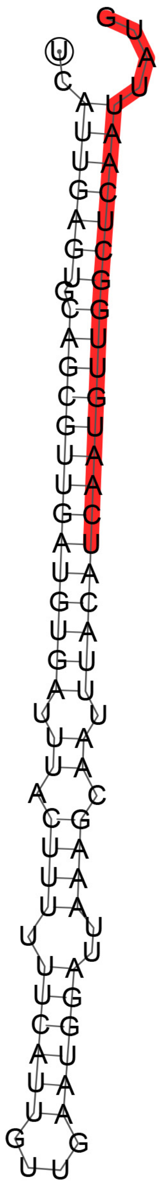

bna-novel _5

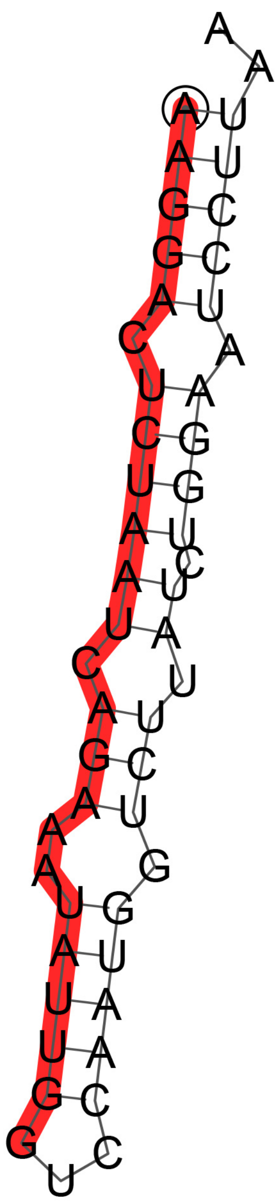

bna-novel _6

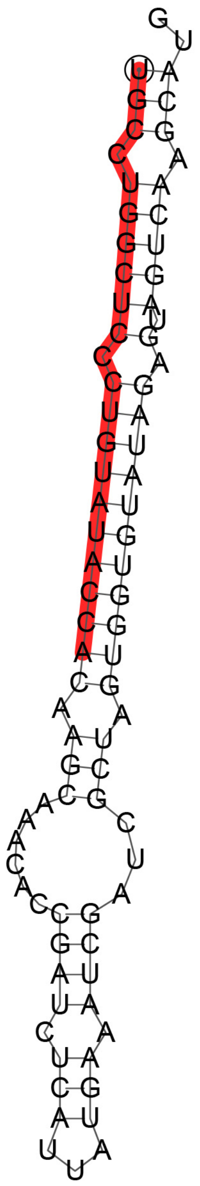

bna-novel _7

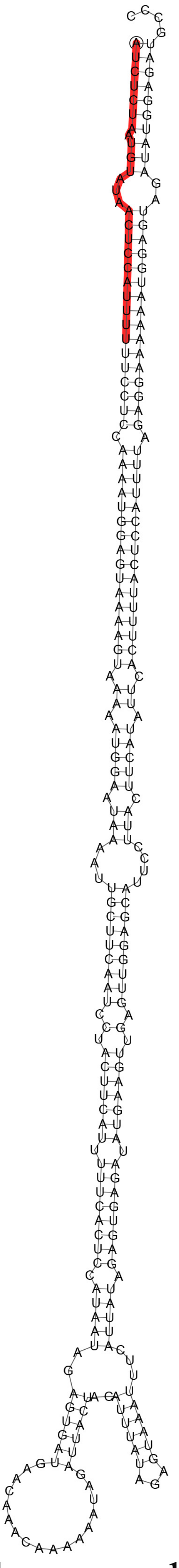

bna-novel _8

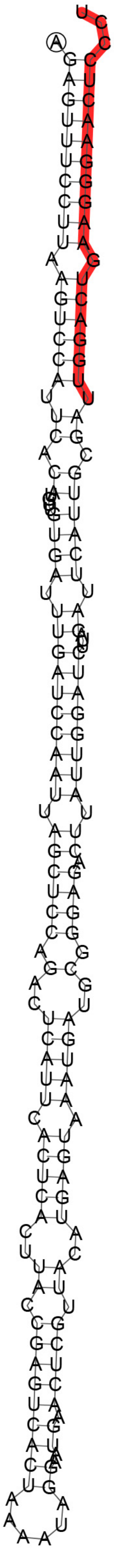

bna-novel_9

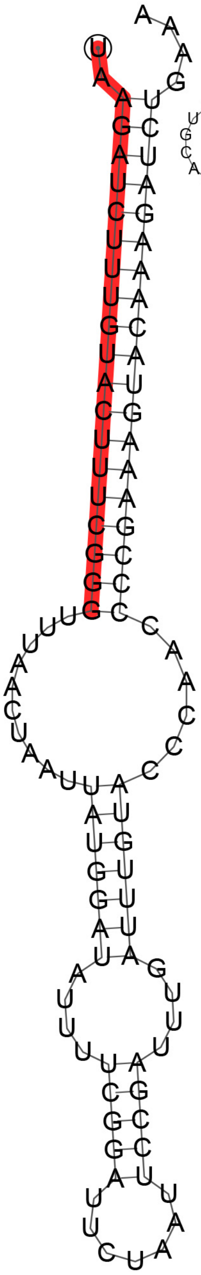

bna-novel_10

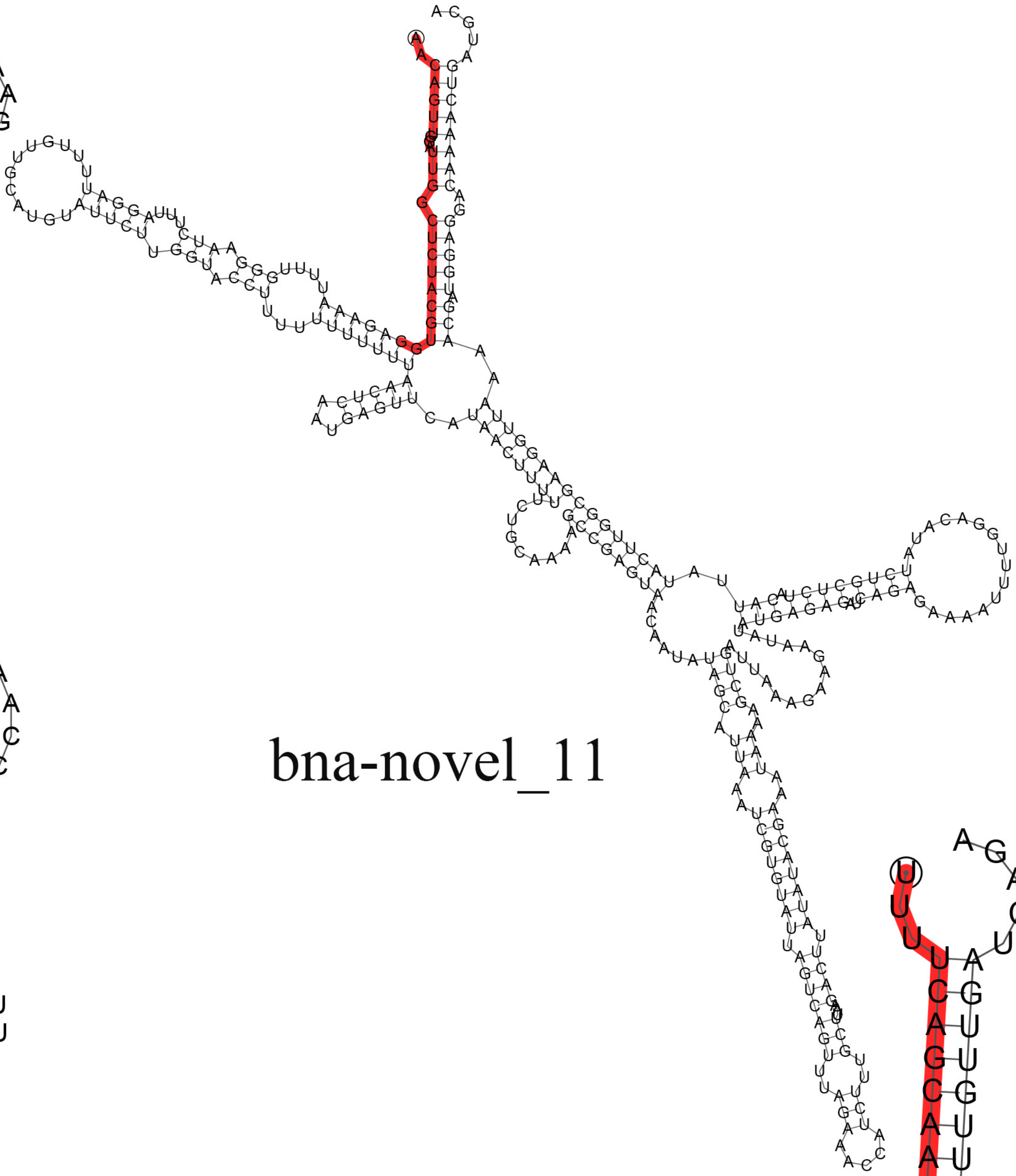

bna-novel_11

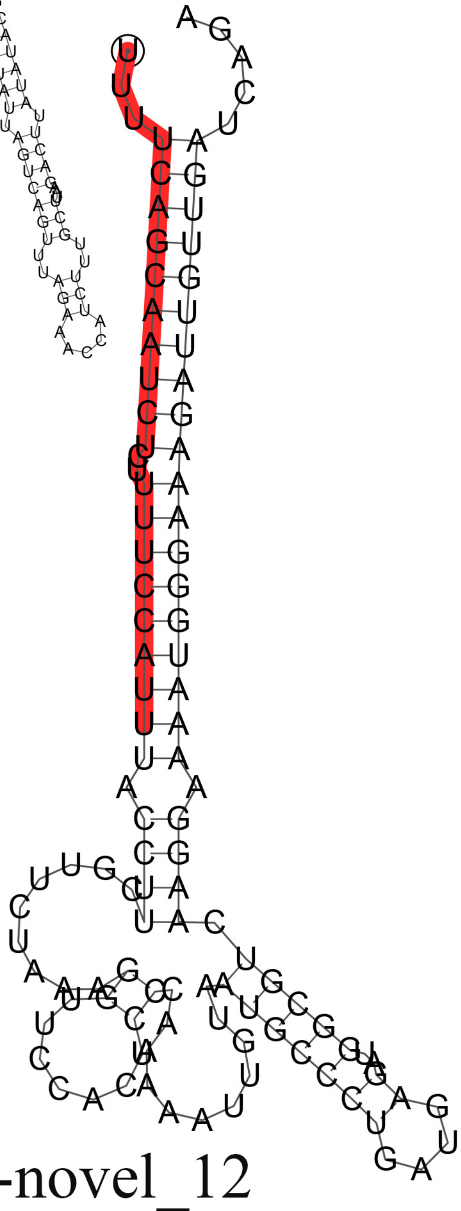

bna-novel_12

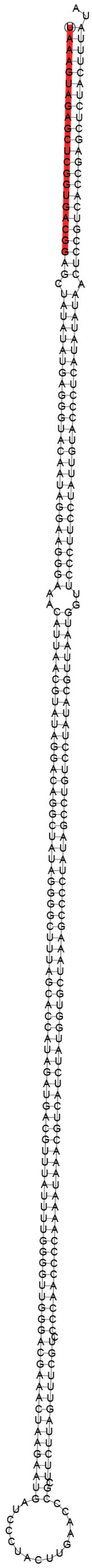

bna-novel_13

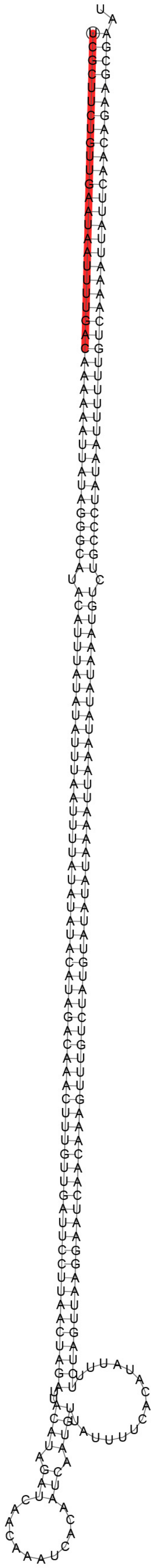

bna-novel_14

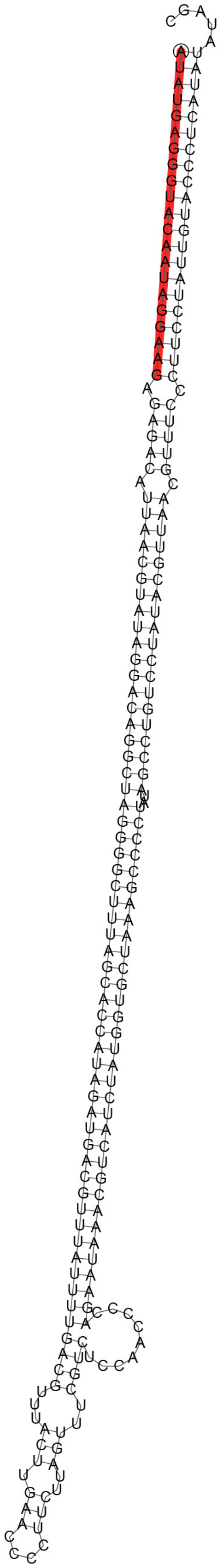

bna-novel_15

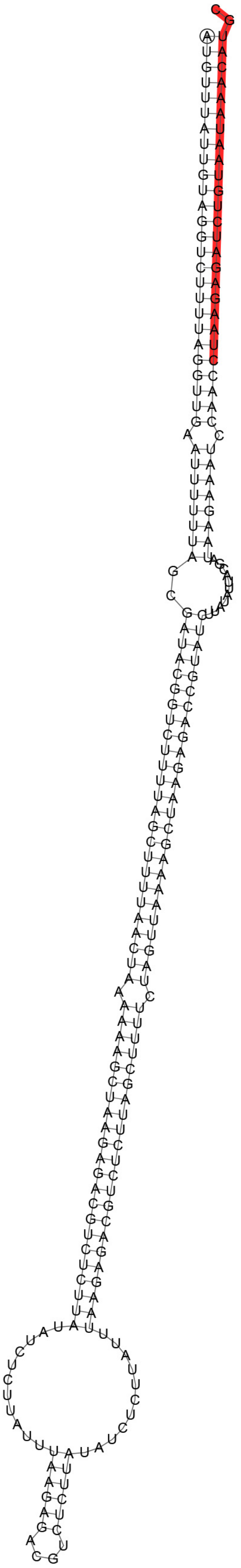

bna-novel_16

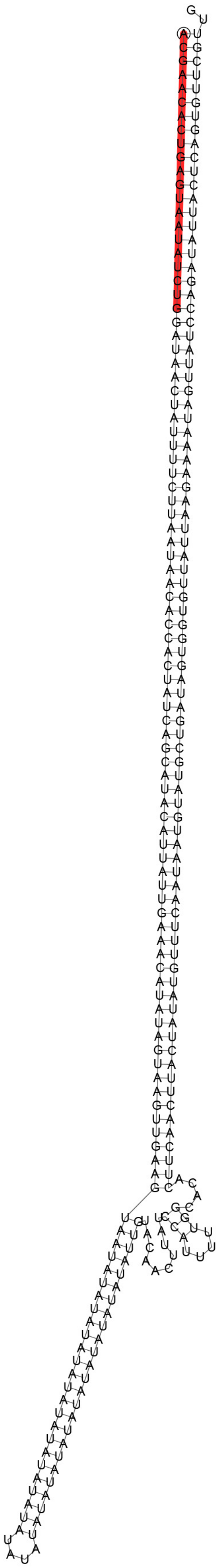

bna-novel_17

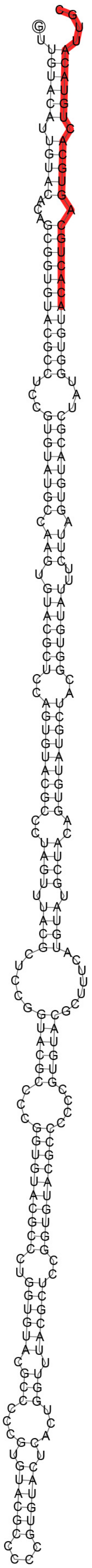

bna-novel_18

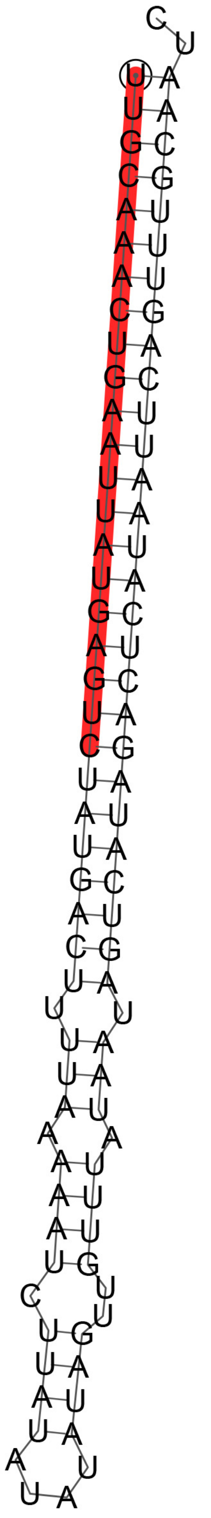

bna-novel_19

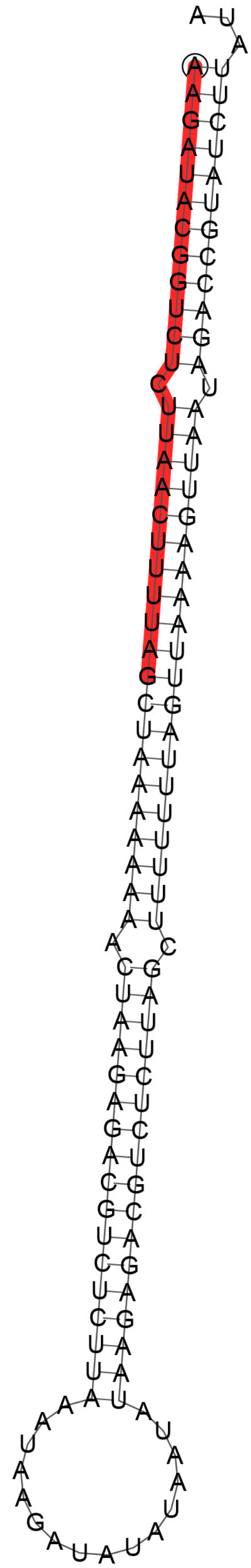

bna-novel 20

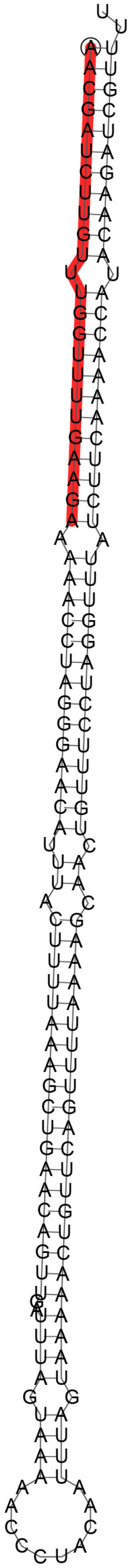

bna-novel_21

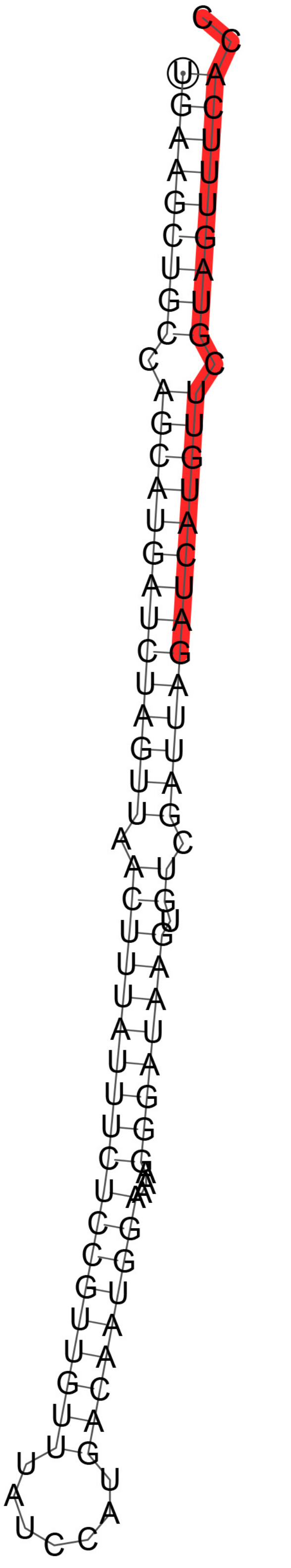

bna-novel_22

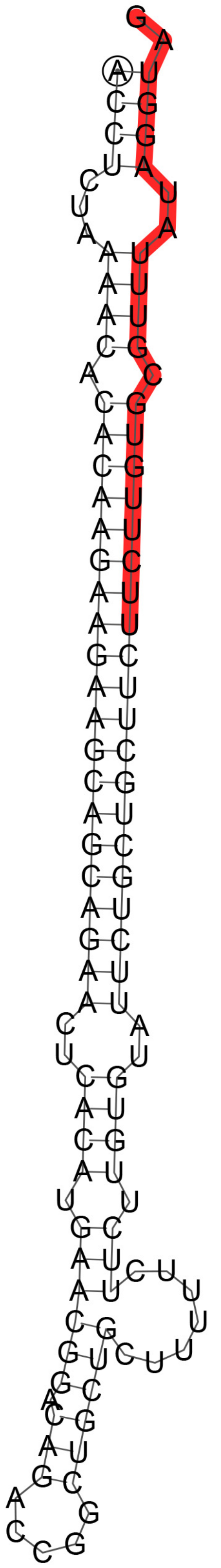

bna-novel_23

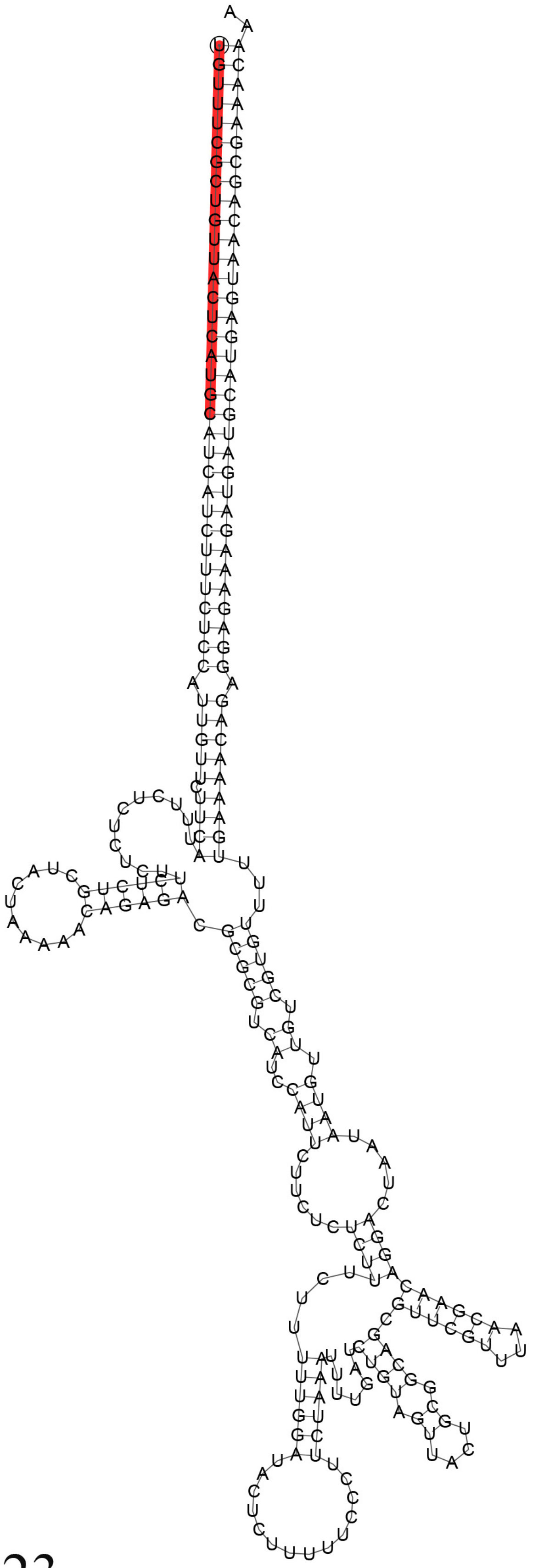

bna-novel_24

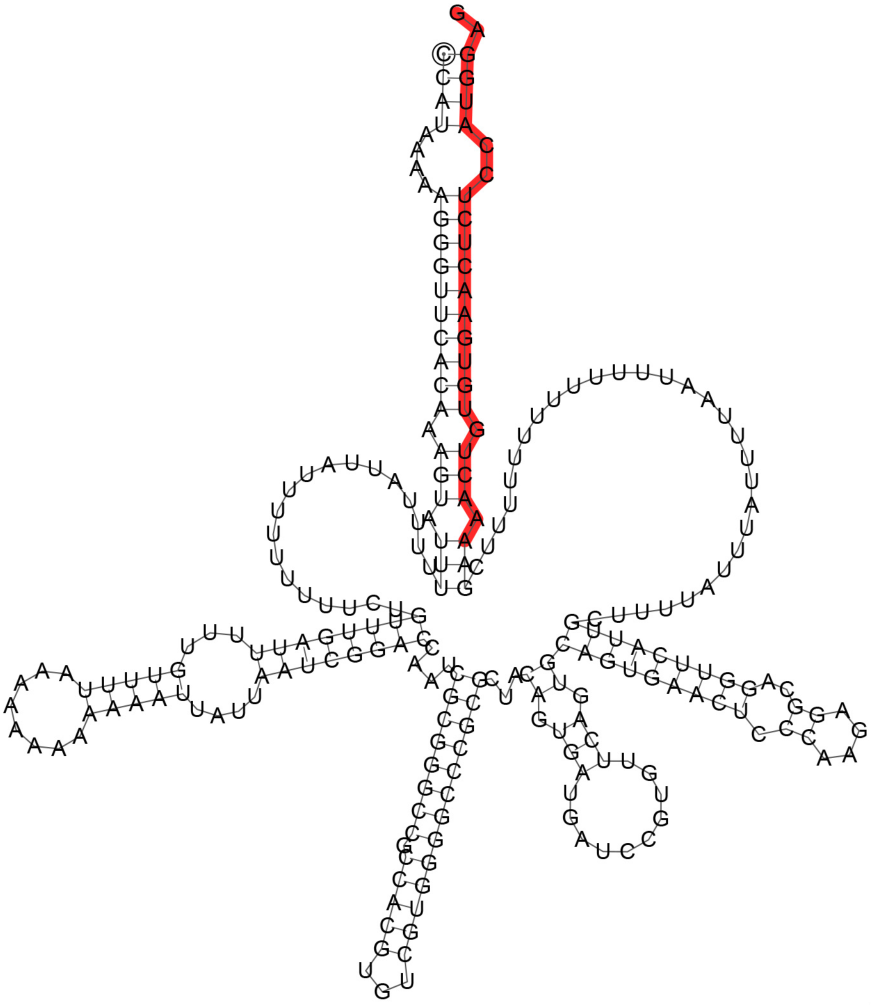

bna-novel_25

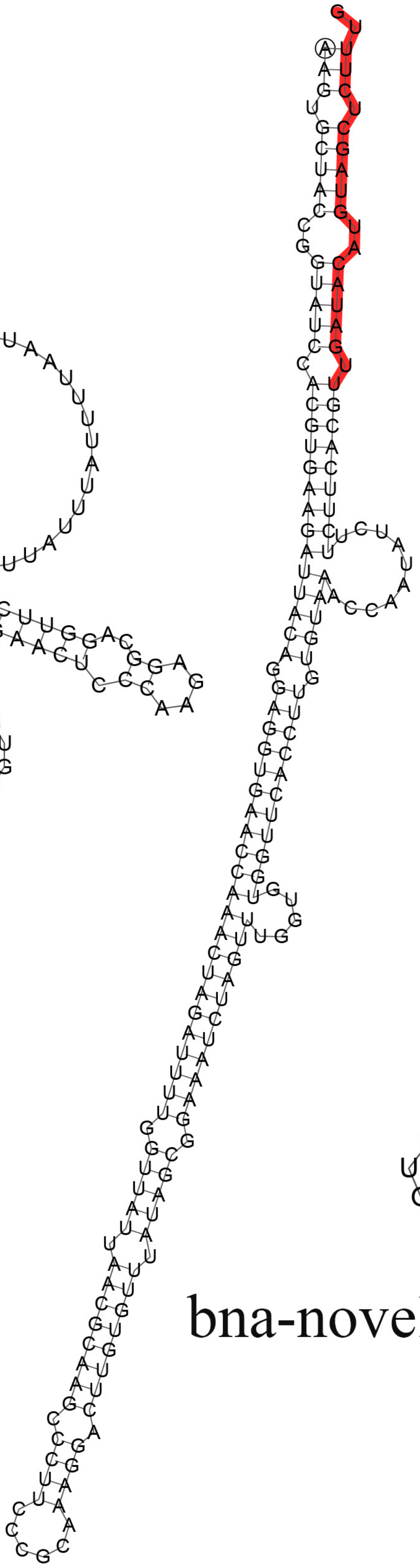

bna-novel_26

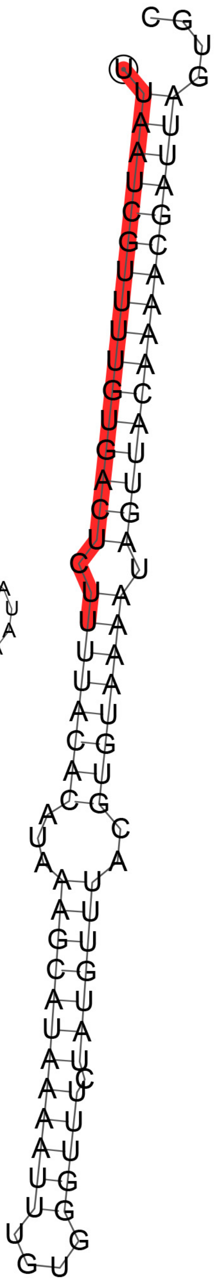

bna-novel_27

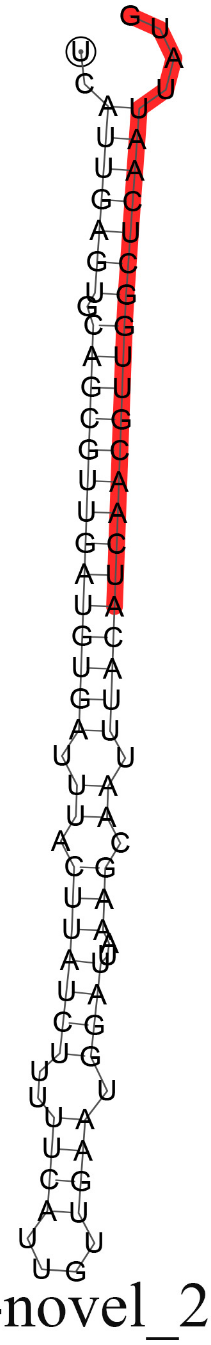

bna-novel_28

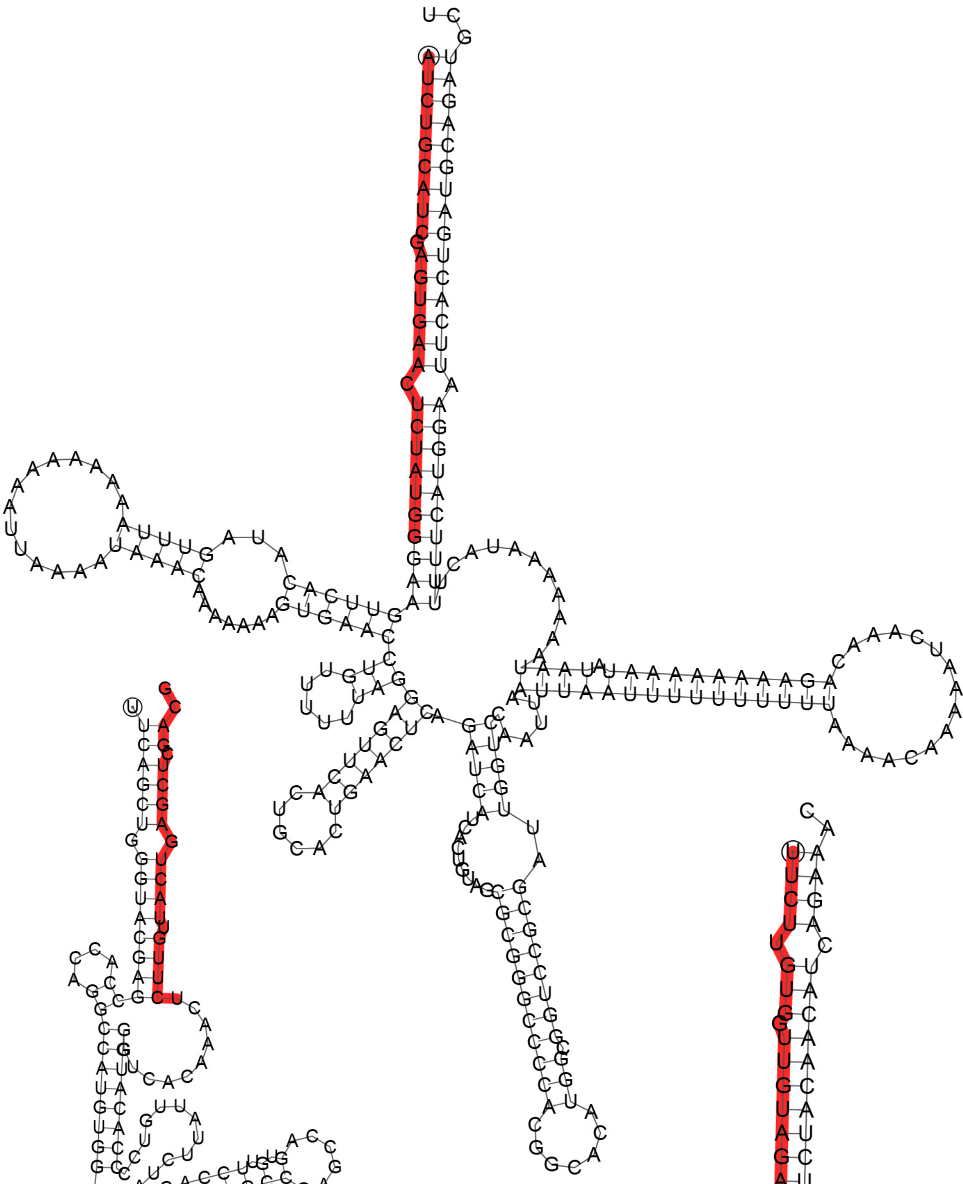

bna-novel_30

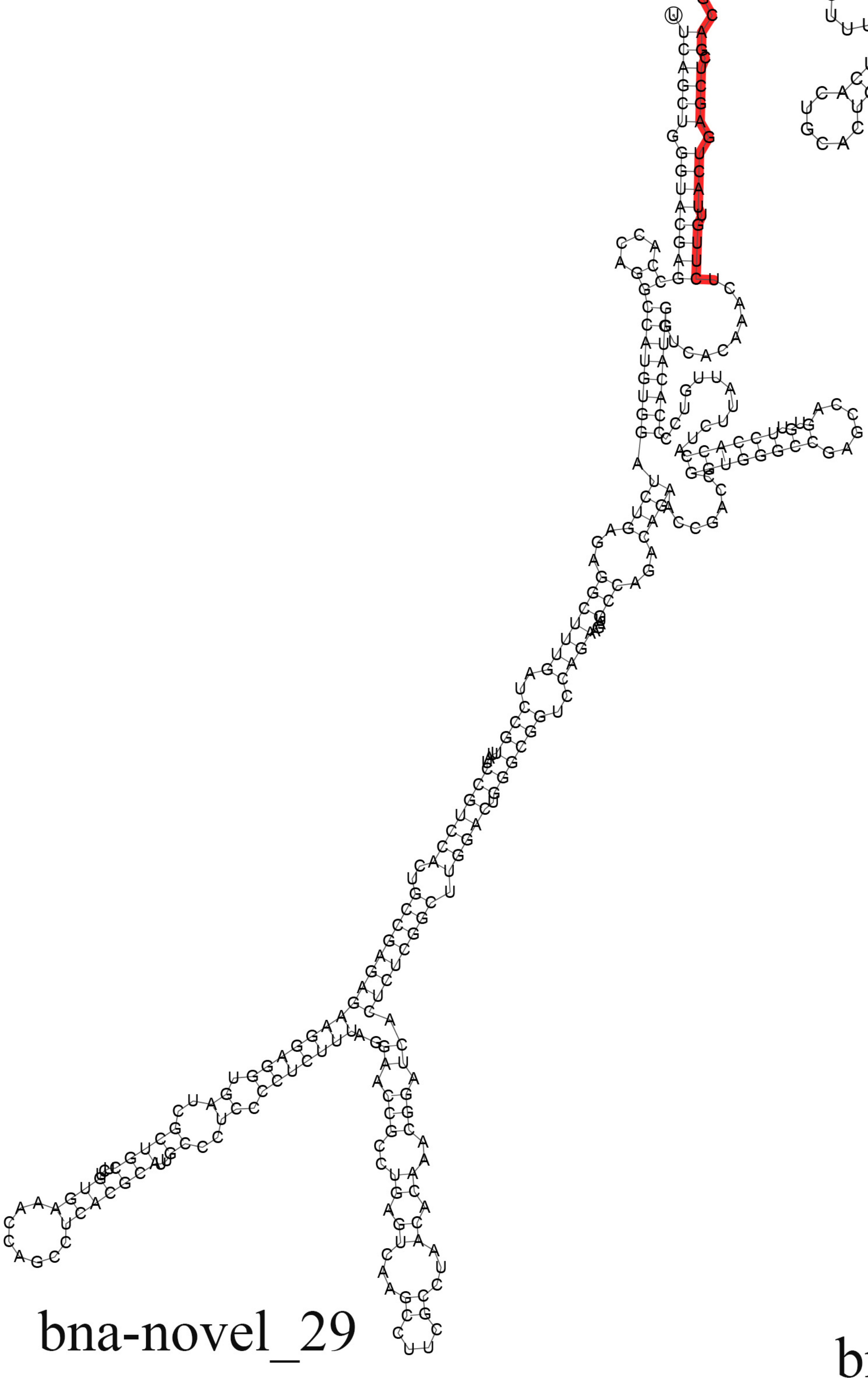

bna-novel_29

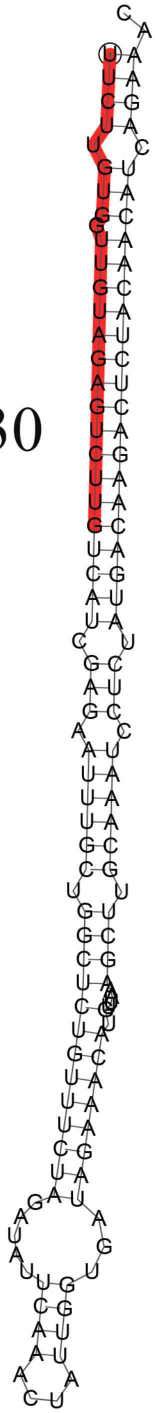

bna-novel_31

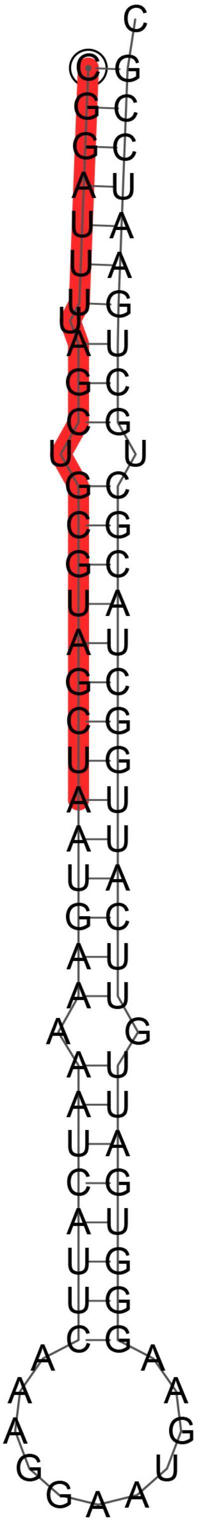

bna-novel_32

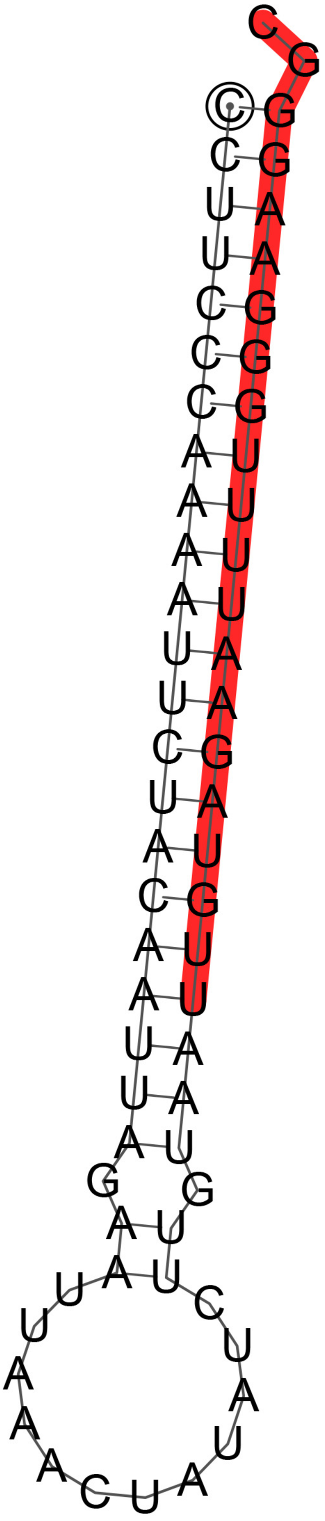

bna-novel_33

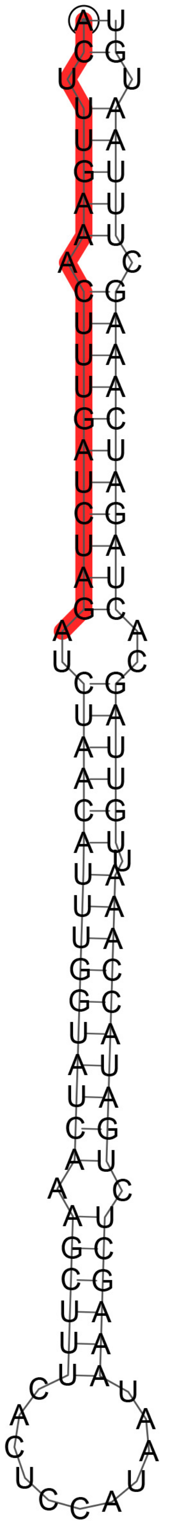

bna-novel_34

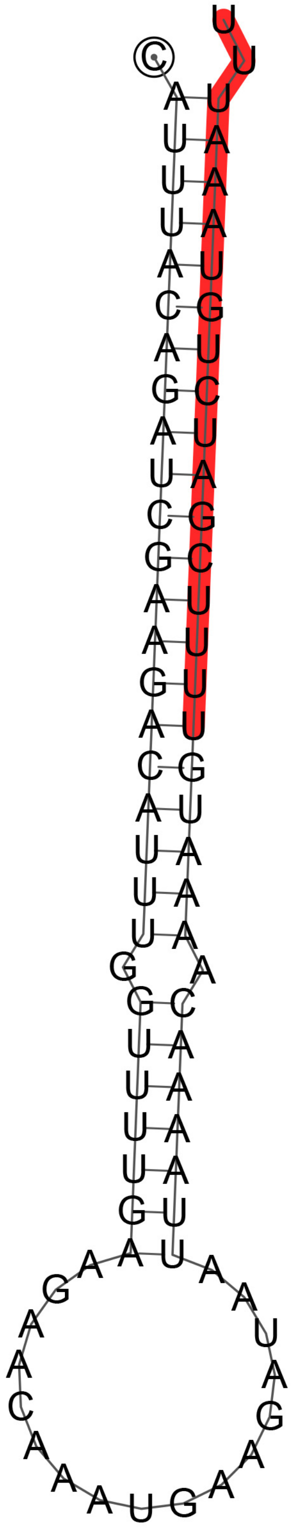

bna-novel_35
